# Landscape of Genetic Alterations Underlying Hallmark Signature Changes in Cancer Reveals *TP53* Aneuploidy–driven Metabolic Reprogramming

**DOI:** 10.1158/2767-9764.CRC-22-0073

**Published:** 2023-02-16

**Authors:** Marni B. McClure, Yasunori Kogure, Naser Ansari-Pour, Yuki Saito, Hann-Hsiang Chao, Jonathan Shepherd, Mariko Tabata, Olufunmilayo I. Olopade, David C. Wedge, Katherine A. Hoadley, Charles M. Perou, Keisuke Kataoka

**Affiliations:** 1 Division of Molecular Oncology, National Cancer Center Research Institute, Tokyo, Japan.; 2 Lineberger Comprehensive Cancer Center, University of North Carolina at Chapel Hill, Chapel Hill, North Carolina.; 3 Department of Medicine, Johns Hopkins School of Medicine, Baltimore, Maryland.; 4 MRC Molecular Haematology Unit, Weatherall Institute of Molecular Medicine, University of Oxford, Oxford, United Kingdom.; 5 Department of Gastroenterology, Keio University School of Medicine, Tokyo, Japan.; 6 Department of Radiation Oncology, Richmond VA Medical Center, Richmond, Virginia.; 7 Department of Radiation Oncology, Virginia Commonwealth University, Richmond, Virginia.; 8 Department of Urology, Graduate School of Medicine, The University of Tokyo, Tokyo, Japan.; 9 Center for Clinical Cancer Genetics & Global Health, University of Chicago School of Medicine, The University of Chicago, Chicago, Illinois.; 10 Manchester Cancer Research Centre, University of Manchester, Manchester, United Kingdom.; 11 Department of Genetics, University of North Carolina at Chapel Hill, Chapel Hill, North Carolina.; 12 Department of Pathology and Laboratory Medicine, University of North Carolina at Chapel Hill, Chapel Hill, North Carolina.; 13 Division of Hematology, Department of Medicine, Keio University School of Medicine, Tokyo, Japan.

## Abstract

The hallmark signatures based on gene expression capture core cancer processes. Through a pan-cancer analysis, we describe the overview of hallmark signatures across tumor types/subtypes and reveal significant relationships between these signatures and genetic alterations. *TP53* mutation exerts diverse changes, including increased proliferation and glycolysis, which are closely mimicked by widespread copy-number alterations. Hallmark signature and copy-number clustering identify a cluster of squamous tumors and basal-like breast and bladder cancers with elevated proliferation signatures, frequent *TP53* mutation, and high aneuploidy. In these basal-like/squamous *TP53*-mutated tumors, a specific and consistent spectrum of copy-number alterations is preferentially selected prior to whole-genome duplication. Within *Trp53-*null breast cancer mouse models, these copy-number alterations spontaneously occur and recapitulate the hallmark signature changes observed in the human condition. Together, our analysis reveals intertumor and intratumor heterogeneity of the hallmark signatures, uncovering an oncogenic program induced by *TP53* mutation and select aneuploidy events to drive a worsened prognosis.

**Significance:**

Our data demonstrate that *TP53* mutation and a resultant selected pattern of aneuploidies cause an aggressive transcriptional program including upregulation of glycolysis signature with prognostic implications. Importantly, basal-like breast cancer demonstrates genetic and/or phenotypic changes closely related to squamous tumors including 5q deletion that reveal alterations that could offer therapeutic options across tumor types regardless of tissue of origin.

## Introduction

Snapshots of gene expression on a genome-wide scale began with the advent of microarray technology. The landmark description of the molecular portraits of breast cancer (1) relying on gene expression robustly predicts overall survival (OS; ref. 2) and metastatic location (3). Since then, >10,000 gene expression signatures have been described to capture complex cancer characteristics, behavior, and etiology. Among them, 50 “hallmark” signatures, nonredundant gene sets generated from the Molecular Signature Database (MSigDB; ref. 4), have been widely used to illustrate the main core of cancer intrinsic and extrinsic pathogenesis including cellular signaling, inflammation, proliferation, and metabolism.

Recent large-scale cancer consortia, including The Cancer Genome Atlas (TCGA; ref. 5), have provided unparalleled, transcriptomic, and genomic sequencing data (6, 7), which have broadened the catalog of somatic alterations underlying cancer development and progression. Particularly, in the 2018 TCGA PanCanAtlas publications, detailed analyses of major pathways, including genomic instability (8), the immune system (9), and oncogenic signaling pathways (10–13), identified the scope of genetic disruptions across cancers. Through these studies, the connection between genetic alterations and transcriptomic changes within a specific pathway or tumor type has been well studied; however, a comprehensive analysis of the relationship between gene expression signatures and genetic alterations has not been described in a systematic, pan-cancer perspective.

These prior efforts have also proposed classifications based on gene expression profiles, identifying novel subtypes within a tumor type or anatomically-related tumor types. Across TCGA compendia, 31 tumor types have been divided into >100 different subtypes that have significantly different genetic and clinical characteristics; however, the landscape of gene expression signatures representing cancer hallmarks, their similarities and differences across tumor types and subtypes, and the specific genetic alterations driving these signatures remain elusive.

Herein, we present a pan-cancer analysis of 48 hallmark signatures across >8,000 cancers from 110 subtypes (of 31 tumor types), exploring their heterogeneity across and within tumor types/subtypes. We investigate the associations of hallmark signatures with 191 somatic alterations, including 59 mutations, 71 arm-level copy-number alterations (CNA), and 61 focal CNAs, employing a rigorous curation of somatic alterations coupled with a robust permutation framework. Among them, we highlight the synergistic relationship of *TP53* and aneuploidy which act in concert to execute an aggressive transcriptional program of high proliferative and high glycolysis expression, especially in squamous cell carcinomas and the basal-like and basal/squamous-like subtypes of breast cancer adenocarcinoma (BRCA) and bladder urothelial carcinoma (BLCA), respectively.

## Materials and Methods

No statistical methods were used to predetermine sample size. The experiments were not randomized and investigators were not blinded to allocation during experiments and outcome assessment.

### TCGA Dataset Preparation

RNA sequencing (RNA-seq) data were downloaded from genomic data commons (GDC) using TCGA PanCancer publication (5) (https://gdc.cancer.gov/about-data/publications/PanCan-CellOfOrigin: EBPlusPlusAdjustPANCAN_IlluminaHiSeq_RNASeqV2.geneExp.tsv), collapsed to the gene level. Tumors were first removed according to TCGA annotation of low-quality samples (ref. 10; *n* = 10,344). Tumor subtypes were determined from tumor-specific TCGA publications including cervical squamous cell carcinoma than endocervical adenocarcinoma (CESC; ref. 14), pheochromocytoma and paraganglioma (PCPG; ref. 15), head and neck squamous cell carcinoma (HNSC; ref. 16), thymoma (THYM; ref. 17), pancreatic adenocarcinoma (PAAD; ref. 18), BLCA (19), kidney renal clear cell carcinoma (KIRC; ref. 20), ovarian serous cystadenocarcinoma (OV; ref. 21), lung squamous carcinoma (LUSC) (22), lung adenocarcinoma (LUAD; ref. 23), skin cutaneous melanoma (SKCM; ref. 24), kidney renal papillary cell carcinoma (KIRP; ref. 25), prostate adenocarcinoma (PRAD; ref. 26), and liver hepatocellular carcinoma (LIHC; ref. 27). For all other subtypes, subtype descriptions from the PanCancer Atlas Oncogenic Signaling publication (10) were utilized (Supplementary Table S1). Tumors represented 31 tumor types (Supplementary Table S3), with 23 harboring subtypes (range: 2–9, median: 4).

### Pan-cancer Hallmark Signature Scores

RNA-seq nontransformed gene expression values of 10,344 TCGA samples were first filtered to the 4,837 genes contained within the 50 hallmark signatures from the h.all.v6.2.symbols.gmt of MSigDB (4). Signature .gmt file and gene expression file were input into single-sample gene set enrichment analysis (ssGSEA 2.0; ref. 28) with default parameters: normalization = “rank,” weight = 0.75, statistic = “area under RES,” output = “NES,” permutations = 1,000, min.overlap = 5, correl.type = “z score.” Each signature score for each sample was then combined for a final matrix and narrowed to those tumors with mutation data (50 signatures by 8,603 samples). We first determined the interquartile range (IQR) of each signature within each tumor subtype, removing KRAS signaling down and spermatogenesis from further analyses due to low expression levels (Supplementary Fig. S2A). Of the remaining 48 signatures, we next measured the extent of overlapping genes for each signature versus each individual other signature by pairwise comparisons utilizing the Jaccard index (Supplementary Fig. S2B; Supplementary Table S2). To evaluate similarity across signatures, we calculated Pearson correlation of each signature to all others using the 8,603 TCGA samples’ ssGSEA output and displayed positive relationships with Benjamini-Hochberg (BH)-adjusted *P* value <1.0 × 10^−50^ (Supplementary Fig. S2B; Supplementary Table S2).

To identify significant groups of well-correlated signatures, TCGA samples were hierarchically clustered using the *hclust* function, dissimilarity calculated as 1 minus the Pearson correlation, and distance calculated by Ward linkage method (Supplementary Fig. S2C). Histogram was divided using *cutree* function, and a cutoff with height = 0.70 was used to identify significant groups. Heatmaps were displayed with *heatmap.2* from the gplots package.

### Clustering of Hallmark Signature Scores Across Subtypes

**FIGURE 1:**
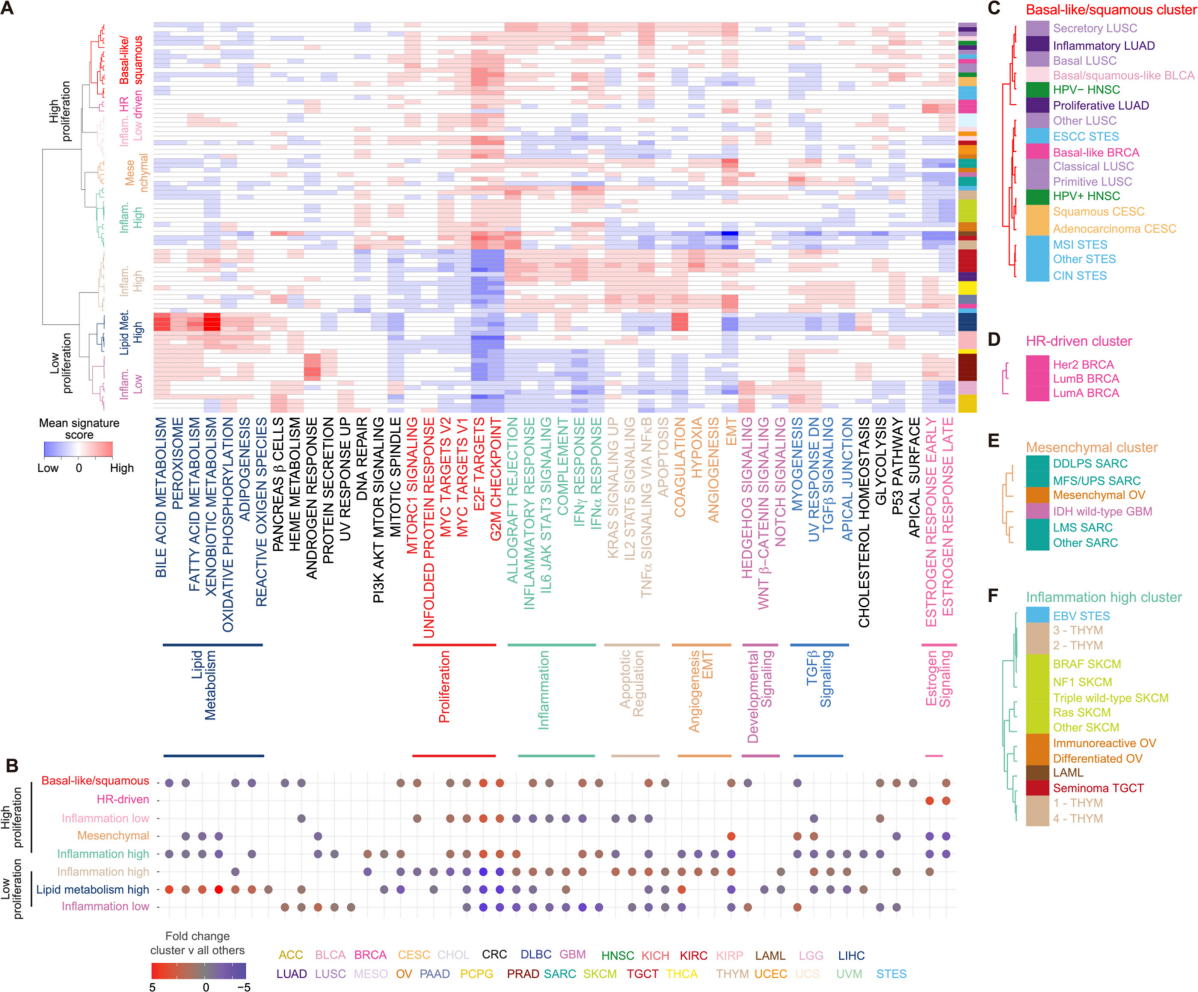
Heterogeneity of hallmark signaling across tumor subtypes. **A,** Mean ssGSEA scores of 48 hallmark signatures in 86 subtypes from 24 tumor types with >100 samples (1–9 subtypes per tumor type, median = 4) are median centered and hierarchically clustered with Pearson correlation and Ward linkage method. Subtypes were defined by a dendrogram height = 0.70, identifying three low proliferation and five high proliferation clusters. Color bar represents each tumor type and is expanded in **C–F**. Red = high expression; blue = low expression. **B,** Significantly altered hallmark signatures for each subtype cluster compared with all other tumor clusters, defined by multiSAM (permutated nonparametric one-sided *t* test), and displayed if *q*-value <1.0 × 10^−4^. Results are ordered on the basis of subtype clusters in **A**. Insets of basal-like/squamous (**C**), HR-driven (**D**), mesenchymal (**E**), and inflammation high clusters (**F**).

To identify significantly associated subtypes by hallmark signatures, mean signature scores of tumors within each tumor subtype or tumor type if no subtypes were available were calculated. Tumor types with at least 100 samples and subtypes with >10 samples were included, leaving 24 of 31 tumor types represented across 86 subtypes (1–9 subtypes/tumor type; Supplementary Table S3). Hierarchical clustering was performed using the *hclust* function, dissimilarity calculated as 1 minus the Pearson correlation, and distance calculated by Ward linkage method (Fig. 1A). The histogram was divided using *cutree* function, and a cutoff with height = 1.6 was used to identify significant groups (Fig. 1C–F). Heatmaps were displayed with *heatmap.2* from the gplots package.

Subtype clusters were then tested as one cluster versus all other clusters using multiSAM (29), a permutated nonparametric *t* test permutated 10,000 times. Fold change is displayed for relationships with FDR < 1.0 × 10^−4^ (Fig. 1B).

### Pan-cancer Mutation Filtering

TCGA PanCancer Atlas GDC mutation maf file (ref. 5; https://gdc.cancer.gov/about-data/publications/PanCan-CellOfOrigin: mc3.v.0.2.PUBLIC.maf.gz) was first downloaded and filtered in two steps: (i) to those tumors containing RNA-seq data, and (ii) removal of those with poor-quality data as annotated in Sanchez-Vega and colleagues (ref. 10; Supplementary Table S6; *n* = 8,603). For tumor suppressors, genes were filtered to the 37 reported tumor suppressors from Hess and colleagues (30). Given the known differences in background mutation rates within subtypes of tumors, not just tumor types, we next identified those tumor suppressors mutated at a strict threshold. First, the number of tumors with both synonymous (variant classifications: 3′Flank, 3′UTR, 5′Flank, 5′UTR, Intron, RNA, Silent) and nonsynonymous mutations were counted within each tumor subtype and each tumor type. A cutoff was determined for each tumor subtype and each tumor type as the third quartile + 3 × IQR of the nonsynonymous mutation count (Supplementary Fig. S3). Only tumor suppressors that had less than 20% silent mutations, were above the outlier threshold, and had at least three mutations were maintained for downstream analyses (Supplementary Table S4). A total of 28 oncogenes were filtered to reported hotspot mutations in Hess and colleagues (30), excluding hotspots from tumor suppressor genes, counted per tumor within each tumor subtype and tumor type, and maintained if the frequency was again above the third quartile + 3 × IQR and had at least three mutations within that tumor subtype or tumor type. This generated a list of 22 oncogenes and 37 tumor suppressors for a total of 151 gene–tumor type combinations for downstream analyses (Supplementary Table S4).

### Pan-cancer CNA Filtering

For focal CNAs, TCGA PanCancer Atlas GDC *in silico* admixture removal (ISAR) corrected SNP whitelisted segmentation file (ISAR_corrected.PANCAN_Genome_Wide_SNP_6_whitelisted.seg) was downloaded and input into Genomic Identification of Significant Targets in Cancer (GISTIC) 2.0 (31) with segment cutoff of 0.10 and broad arm alteration ≥50% for tumor subtypes and tumor types. GISTIC focal CNAs from both the subtype and tumor type analysis were then filtered to *q* ≤ 0.01 and a width of 250,000 bases. Second, all significant segments were collapsed to overlapping ranges using *findOverlaps*, permitting any base overlap. Finally, these common focal segments were mapped to the gene level with the closest cancer-related gene per OncoKB (32). This generated a list of 61 focal alterations from 29 tumor types with a total of 226 focal alteration–tumor type combinations (Supplementary Table S3).

Binary focal CNA calls were used from the GDC PanCan Atlas (5) matrix ISAR_GISTIC.all_data_by_genes.txt.gz and limited to samples with both mutation and RNA-seq data available (*n* = 8,264) and the 61 focal CNAs from GISTIC analysis.

For arm-level CNAs, GISTIC 2.0 was run with segment cutoff of 0.10 but a broad arm alteration ≥98%. GISTIC broad alterations from both the subtype and tumor type analysis were then filtered to a *q* ≤ 1.0 × 10^−3^. This generated a list of 71 significant arm-level CNAs across 30 tumor types for a total of 487 arm-level CNA-tumor type combinations (Supplementary Table S3).

Arm-level CNA calls for each tumor were downloaded from TCG PanCan Atlas GDC: PANCAN_ArmCallsAndAneuploidyScore_092817.txt and limited to samples with both mutation and RNA-seq data available (*n* = 8,231). This matrix was expanded so that each column represented one chromosome arm either amplified or deleted, and calls were converted to binary value (e.g.: for 1p Amp., 0 = not amplified; 1 = amplified and for 1p Del, 0 = not deleted; and 1 = deleted).

### Permutation Framework for Identifying Relationships Consistent Across Tumor Types

**FIGURE 2:**
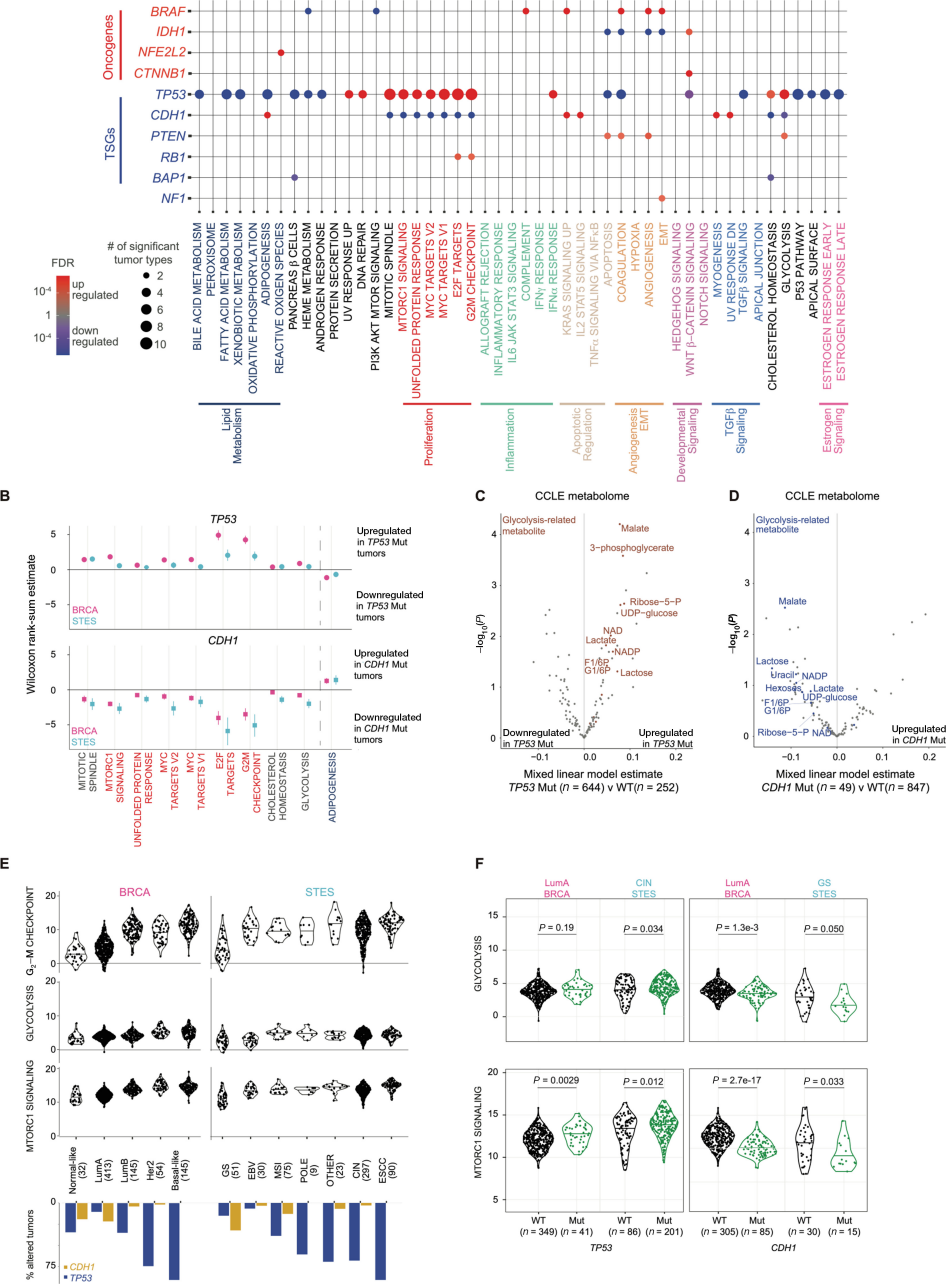
Mutation hallmark signature relationships across cancer. **A,** Permutation test resulted in 65 significant signature–mutation relationships consistently observed across tumor types (representing 219 signature–mutation–tumor type relationships with Wilcoxon rank-sum test). Dot size and color represent the number of significant tumor types and BH-adjusted FDR (red = upregulated signature with mutation; blue = downregulated signature with mutation, purple = upregulated and downregulated in different tumor types), respectively. **B,** Wilcoxon rank-sum estimates of TP53 (circle) wild-type versus mutant and CDH1 (square) mutant versus wild-type tumors across BRCA (pink) and STES (blue) tumors. Error bars indicate 95% CIs. Linear mixed model testing 225 metabolites from 896 CCLE cell lines from 39 lineages compared across TP53 (**C**) and CDH1 (**D**) wild-type versus mutant tumors with lineage as a random effect. Colored dots and labels indicate 12 metabolites involved in the glycolysis pathway as annotated from the Human Metabolite Database. **E,** Kernel density plot of signature scores for G_2_–M checkpoint, MTORC1 signaling, and glycolysis in BRCA and STES. Bar graph shows the percent of tumors harboring TP53 (blue) and CDH1 mutations (gold) within each subtype. **F,** Kernel density plot of signature scores for G_2_–M checkpoint, MTORC1 signaling, and glycolysis within LumA BRCA, CIN STES, and GS STES, comparing TP53 (left) or CDH1 (right) wild-type versus mutant tumors. Wilcoxon rank-sum test *P* values are reported.

**FIGURE 3:**
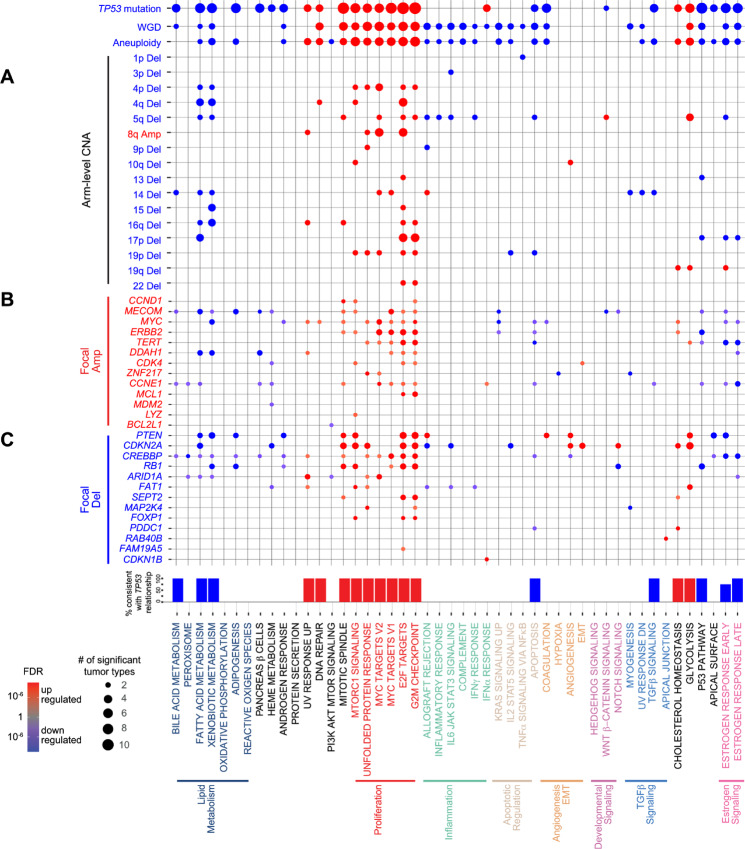
CNAs drive similar hallmark signatures and reflect signature–*TP53* mutation relationship. **A,** A total of 73 significant signature–arm-level CNA relationships were consistently observed across tumor types (representing 163 signature–arm-level CNA–tumor type relationships with Wilcoxon rank-sum test ≤1.0 × 10^−7^). Consistent signature relationships with TP53 mutation, WGD, and aneuploidy score (Wilcoxon rank-sum *P* value ≤1.0 × 10^−3^) displayed above. Dot size and color represent the number of significant tumor types and BH-adjusted FDR (red = upregulated signature with CNA; blue = downregulated signature with CNA), respectively. Percent of arm-level relationships consistent with TP53 mutation displayed for each signature. A total of 182 significant signature–focal CNA relationships were consistently observed across tumor types with 13 amplifications (**B**) and 13 deletions (**C**; representing 462 signature–focal CNA–tumor type relationships with Wilcoxon rank-sum test ≤1.0 × 10^−3^). Dot size and color represent the number of significant tumor types and BH-adjusted FDR (red = upregulated signature with CNA; blue = downregulated signature with CNA), respectively.

**FIGURE 4:**
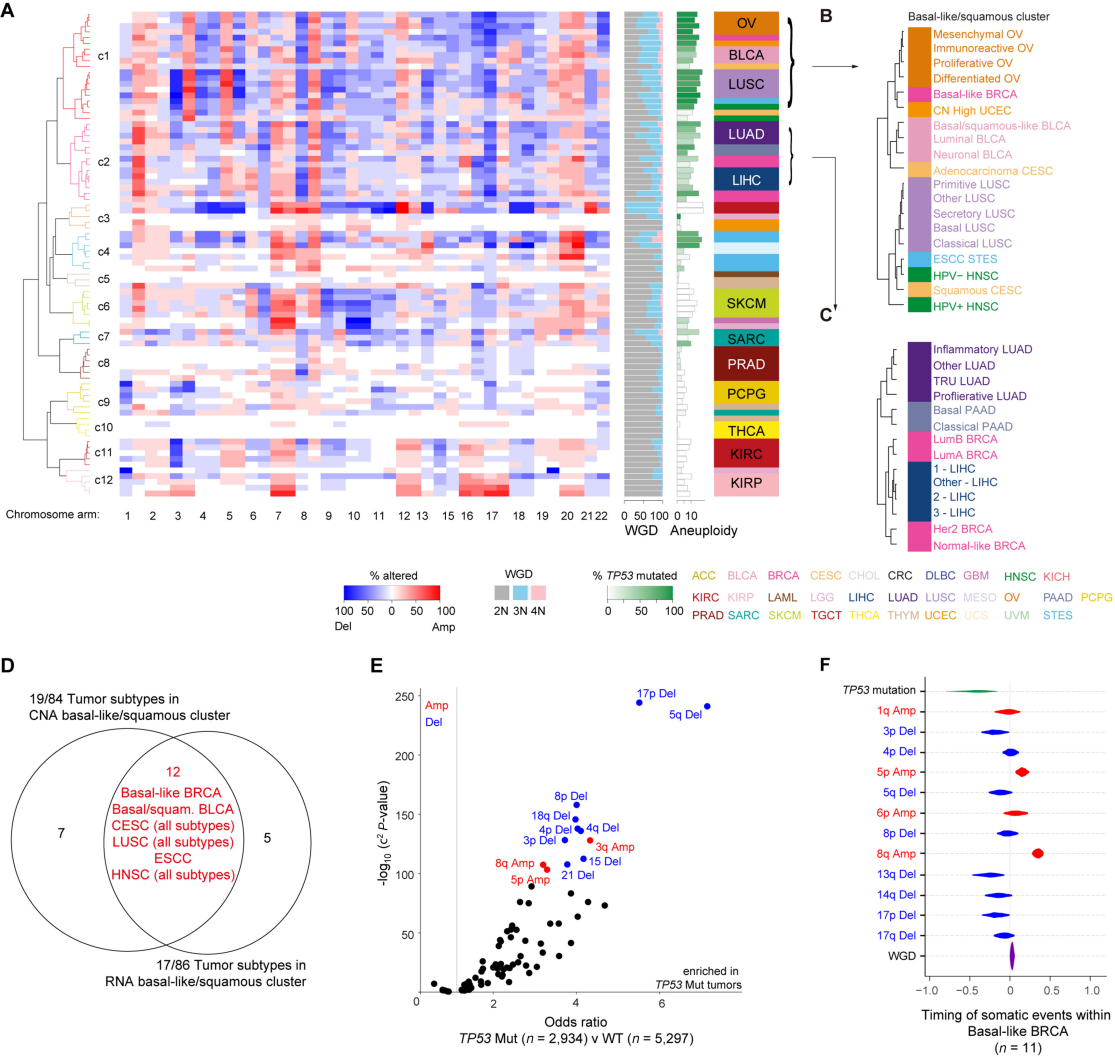
Arm-level CNAs recapitulate hallmark cluster across cancer subtypes, occurring prior to WGDs. **A,** Percent of arm-level CNAs of 39 chromosome arms in 84 subtypes from 24 tumor types with at least 100 samples are median centered and hierarchically clustered with Pearson correlation and Ward linkage method. Color bar represents each tumor type. Red = percent tumors amplified; blue = percent tumors deleted. Side bar indicates the percent with WGD (left) and TP53 mutation (right), and average aneuploidy score (right) within a given tumor subtype: gray = 2N; sky blue = 3N or 1 WGD; light pink ≥4N or ≥2 WGD. Insets of high aneuploidy clusters with high (**B**) and moderate (**C**) frequencies of TP53 mutation. **D,** Overlap of tumor subtypes between hallmark signature squamous cluster and arm-level CNA squamous cluster (Fisher exact *P* = 1.1 10–6). **E,***χ*^2^ test of TP53 wild-type versus mutated samples for each arm-level deletion and amplification. Blue = deletion; red = amplification. **F,** Timing of somatic events plotted as the 95% CIs based on WGS data from basal-like BRCA tumors which harbor TP53 mutation, WGD, and 5q deletion (*n* = 11). Additional enriched CNAs within these tumors are also displayed.

Somatic alterations were first filtered to those present in at least two tumor types (mutations = 28 genes, 120 gene–tumor type combinations; focal CNAs = 61 focal CNAs, 223 gene–tumor type combinations; arm-level CNAs = 58, 474 arm-level CNA–tumor type combinations; Supplementary Fig. S1E). For each tumor type–somatic alteration pair, Wilcoxon rank-sum test was performed across the 48 hallmark signatures (Supplementary Table S6). The number of significant tumor types for each somatic alteration–signature pair and an increase in signature and a decrease in signature (mutation: *P* ≤ 1.0 × 10^−3^; focal CNA: *P* ≤ 1.0 × 10^−3^; arm-level CNA: *P* ≤ 1.0 × 10^−7^) were counted. We filtered to only those relationships significant in more than one tumor type. Then, if at least one tumor type was significantly altered in either direction, permutation testing was applied to minimize both confounding and false-positive relationships for each fold change separately: First, tumor types with the somatic alteration from the above filtering had mutation label randomized 1,000,000 times, and Wilcoxon rank-sum test *P* values and estimates were recorded (Supplementary Fig. S1F). Then, the number of significant tumor types with estimates in the same direction as the one being tested were calculated at the above predefined *P*-value thresholds and compared with the originally detected number of significant tumor types. False positives were counted to define a FDR, and FDRs were adjusted by the BH method for multiple testing corrections (Supplementary Table S7). Relationships with BH-adjusted FDR for mutations and focal CNAs at 1.0 × 10^−4^ and arm-level CNAs at 1.0 × 10^−7^ are displayed (Supplementary Fig. S1G; Figs. 2A, 3A, and 4A).

### Identification of Subtype-independent Signature–Somatic Alteration Relationships

For those tumor types containing subtypes, generalized linear model (GLM) was performed comparing signature score with somatic alteration with subtype as a covariate within each tumor type. GLM estimates and BH-adjusted *P* values were recorded (Supplementary Table S9), and a BH-adjusted *P* value <0.050 was considered significant.

### Cell Line Metabolomics and Hallmark Signature Validation

Genomics data from DepMap project (33) including RNA-seq (CCLE_expression_19Q3.csv; 1,163 cell lines), mutation data (CCLE_mutations_19Q3.csv; 1,666 cell lines), and the annotation file (CCLE_sample_info.csv; 1,736 cell lines) were downloaded at the 19Q3 freeze. Metabolomics data (34) for 928 cell lines across 225 metabolites were downloaded and overlapped with the The Cancer Cell Line Encyclopedia (CCLE) mutation data (896 cell lines in both datasets). Metabolites of mutated versus wild-type cell lines were compared by *lmer* from the *lme4* package with cell lineage as a covariate, including only those tumor types that were significant in TCGA data. For *TP53* mutation, this includes BRCA, HNSC, LIHC, LUAD, and stomach and esophageal carcinoma (STES). For *CDH1* mutation data, this is BRCA and STES. Calculated t-statistic and BH-adjusted *P* value are reported (Fig. 2C and D). Metabolites were curated using the Human Metabolome Database (35) to define the metabolic pathway involved.

### Arm-level CNA Clustering

**FIGURE 5:**
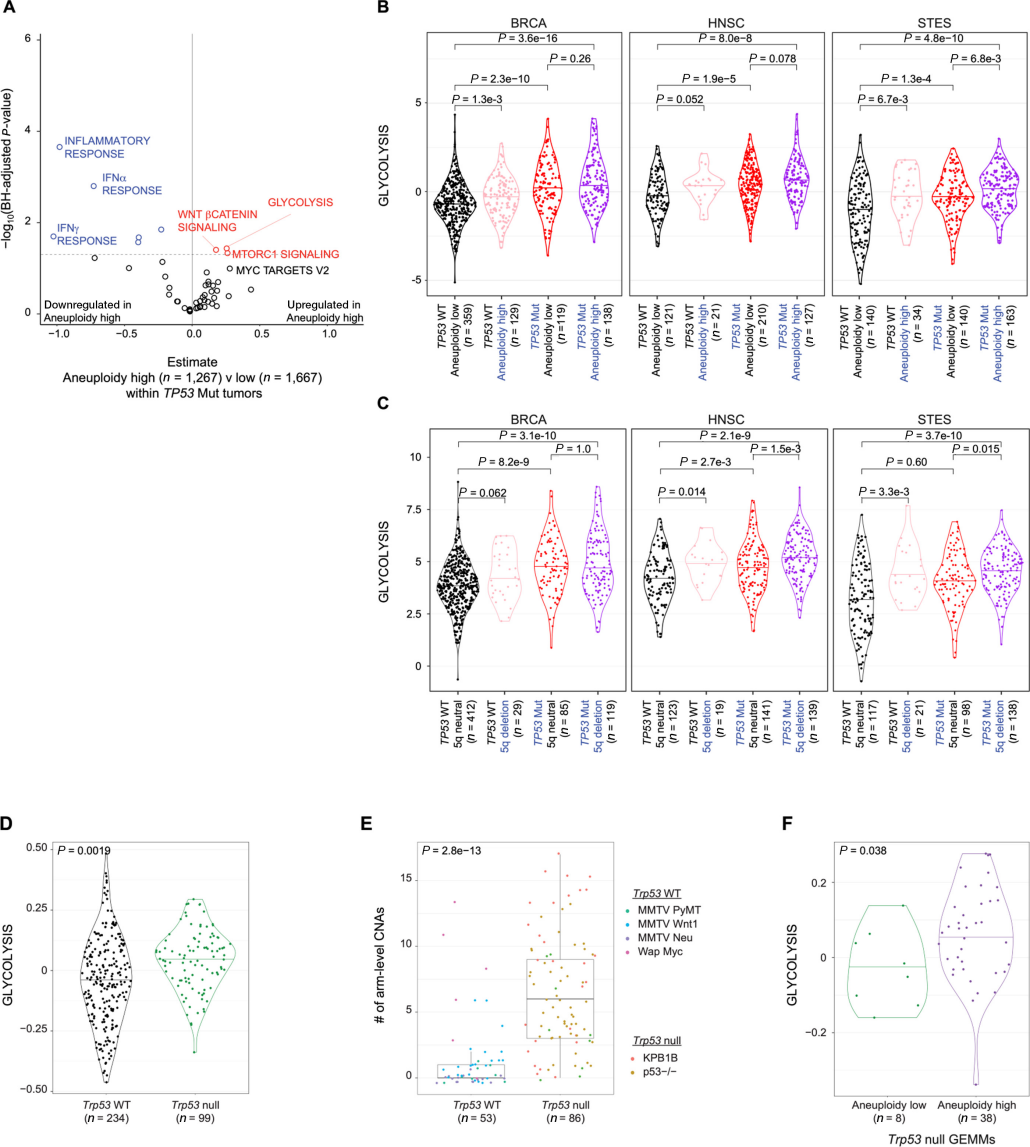
Consecutive arm-level CNA alterations within TP53 mutated tumors. **A,** Effect of aneuploidy high (>15) versus low (≤15) on each hallmark signature within the 12 basal-like/squamous subtypes. GLM was used with subtype as a covariate. *x* axis indicates the GLM estimate, *y* axis shows the BH-adjusted *P* value. Kernel density plot of glycolysis signature comparing TP53 wild-type versus mutant tumors with low versus high aneuploidy (**B**) and 5q neutral versus deletion (**C**). Wilcoxon rank-sum *P* values are reported for each comparison. **D,** Kernel density plot of glycolysis signature comparing *Trp53* wild-type versus null GEMMs. **E,** Boxplot demonstrating median, upper, and lower quantiles of arm-level CNAs in *Trp53* wild-type versus null GEMMs, defined as amplification or deletion affecting >50% of each chromosome arm. **F,** Kernel density plot of glycolysis signature comparing low (<2 CNAs) versus high (≥2 CNAs) aneuploidy within *Trp53*-null GEMMs. **A**–**C,** GLM *P* values are reported using mouse strain as a covariate.

To explore the consistency of arm-level CNAs across tumor subtypes, binary arm-level amplifications and deletions were utilized. For each tumor subtype at each chromosome arm, the percent of tumors with arm-level amplification and arm-level deletion was calculated. Next, the higher percentage alteration (amplification vs. deletion) was maintained for that chromosome arm. Percentage of deletions was multiplied by −1, and a matrix of arm-level CNAs versus tumor subtypes was generated (39 chromosome arms by 104 subtypes). Subtypes were filtered to those tumor types with at least 100 samples and subtypes with at least 10 samples, leaving 84 tumor subtypes from 24 tumor types for clustering [thyroid carcinoma (THCA) Other and CRC Other were excluded from CNA cluster because <10 tumors from these subtypes had aneuploidy data reported]. Finally, hierarchical clustering was performed with the *hclust* function, calculated as 1 minus the Pearson correlation, and distance calculated by Ward linkage method. The histogram was divided using *cutree* function, and a cutoff with height = 1.1 was used to identify significant groups. Heatmaps were displayed with *heatmap.2* from the gplots package (Fig. 5A).

### Comparison of Focal and Arm-level CNAs

To evaluate the co-occurrence of focal CNAs and arm-level CNAs, the 61 focal CNAs for each gene (28 focal amplifications; 33 focal deletions) within each tumor type (223 focal CNA–tumor type combinations) were compared with the arm-level CNA of that gene within each tumor (i.e., MYC focal amplification and 8q arm-level amplification within BRCA). Then, the percent of tumors with the arm-level CNA altered in the same direction as the focally altered gene for that gene–tumor type combination was calculated (Supplementary Fig. S5C and S5D).

### Timing Analysis

TCGA whole-genome sequencing (WGS) for BRCA was downloaded from GDC (7) and limited to White and Black ethnic groups only (*n* = 76). The Battenberg algorithm (36) was used to call CNAs based on WGS data. Mutations were also called using MuTect (v1.1.7; https://software.broadinstitute.org/cancer/cga/mutect) and Strelka (v1.0.13; https://github.com/Illumina/strelka), and the intersection was annotated using ANNOVAR (37). On the basis of cancer cell fraction (CCF), variants were then classified as clonal (CCF = 1) and subclonal (CCF < 1) using DPClust (https://github.com/Wedge-lab/dpclust). CCF of CNAs was obtained from Battenberg. Whole-genome duplication (WGD) was assigned to tumors with an average ploidy ≥3. To select enriched CNA events, the frequency of each CNA was obtained in the dataset and based on a permutation test (*n* = 1,000), which was followed by an FDR-based multiple-testing adjustment step. Those with a frequency above the random background rate were selected. Tumors with *TP53* mutation, 5q deletion, and WGD were identified (*n* = 11), and all observed somatic events were ordered on the basis of CCF per tumor. The Plackett-Luce model for ordering partial rankings (https://github.com/hturner/PlackettLuce) was implemented to infer the order of events based on the ordering matrix of the entire dataset. This analysis was repeated 1,000 times to obtain the 95% confidence interval (CI) of the timing estimate of each event. 95% CI is plotted for these features (Fig. 5F).

### Breast Cancer Mouse Model Analysis of Gene Expression and CNAs

Gene expression microarray data were downloaded from the University of North Carolina microarray database (https://genome.unc.edu) and are also available at GSE42640 (38), GSE122076 (39), and GSE107432 (40), probes were filtered by a lowest normalized intensity in sample and control >10, normalized to the log_2_ ratio of Cy5 tumor/Cy3 control, and collapsed by median to gene (Supplementary Table S12). Gene expression was median centered across the dataset. Mouse homologs of the human hallmark signature dataset were mapped using *homologene*, and the mean of genes within each signature was calculated.

CN array CGH data were downloaded from GSE52173 (41) and GSE122076 (39) and segmented with SWITCHdna (42) with *F* = 12 and α = 20 as described previously (41). log_2_ copy-number segments >0.1 were considered amplified, and <−0.1 as deleted. For each genetically engineered mouse model (GEMM) tumor, arm-level CNAs were determined by calculating the percentage of the arm altered and considered as arm-level altered if >50% of the arm had a copy-number change in the same direction. Aneuploidy was calculated as the sum of arms amplified or deleted.

### Molecular Taxonomy of Breast Cancer International Consortium Dataset Analysis

Molecular Taxonomy of Breast Cancer International Consortium (METABRIC) gene expression data (43) were first downloaded from https://www.cbioportal.org/study/clinicalData?id=brca_metabric. The downloaded matrix was narrowed to genes overlapped with the hallmark signature scores and input into ssGSEA with the same parameters as above (1,992 samples × 4,350 genes). ssGSEA output for each hallmark signature was then collapsed to one matrix (1,992 samples × 50 hallmark signatures).

METABRIC mutation data (44) were also downloaded from https://www.cbioportal.org/study/clinicalData?id=brca_metabric. Mutations were filtered to genes significant in the significantly mutated gene test for BRCA specifically (*TP53, CDH1, GATA3, MAP3K1,* and *PIK3CA*) and to nonsynonymous mutations. Next, Wilcoxon rank-sum test comparing each hallmark signature with mutated versus wild-type tumors for each gene was performed. Finally, Wilcoxon rank-sum test results were compared with those from TCGA BRCA cohort (Supplementary Table S13; Supplementary Fig. S8B and S8C).

METABRIC focal CNA data (43) were downloaded from https://www.cbioportal.org/study/clinicalData?id=brca_metabric. For those genes significant in TCGA GISTIC analysis of BRCA and BRCA subtypes (*ERBB2* Amp, *MYC* Amp, *CCND1* Amp, *PTEN* Del, and *RB1* Del), focal CNA data from METABRIC were compared with hallmark signatures using Wilcoxon rank-sum test. The METABRIC estimates were compared with TCGA estimates for each focal CNA–signature pair (Supplementary Table S13; Supplementary Fig. S8D).

METABRIC segmentation data (43) generated with circular binary segmentation method for 995 samples were input into GISTIC 2.0 with the same criteria used in TCGA analysis (segment cutoff of 0.1 and broad arm alteration ≥50%). GISTIC output for each arm were converted to binary arm level data with >0.2 considered as amplified and <−0.2 as deleted. Next, Wilcoxon rank-sum test for those arm-level CNAs tested in TCGA BRCA cohort were compared with hallmark signature scores (4p Del, 4q Del, 5q Del, 8q Amp, 13q Del, 14q Del, 15q Del, 17p Del, and 19p Del). The METABRIC estimates were compared with TCGA estimates for each arm-level CNA–signature comparison (Supplementary Table S13; Supplementary Fig. S8E).

METABRIC clinical data were also downloaded from https://www.cbioportal.org/study/clinicalData?id=brca_metabric and merged with ssGSEA hallmark signature scores and *TP53* mutation data (*n* = 1,826). Glycolysis signature from ssGSEA Hallmark calls were converted to a binary score by first calculating the quartiles for the METABRIC dataset and next defining high glycolysis as tumors with expression ≥ third quartile.

**FIGURE 6:**
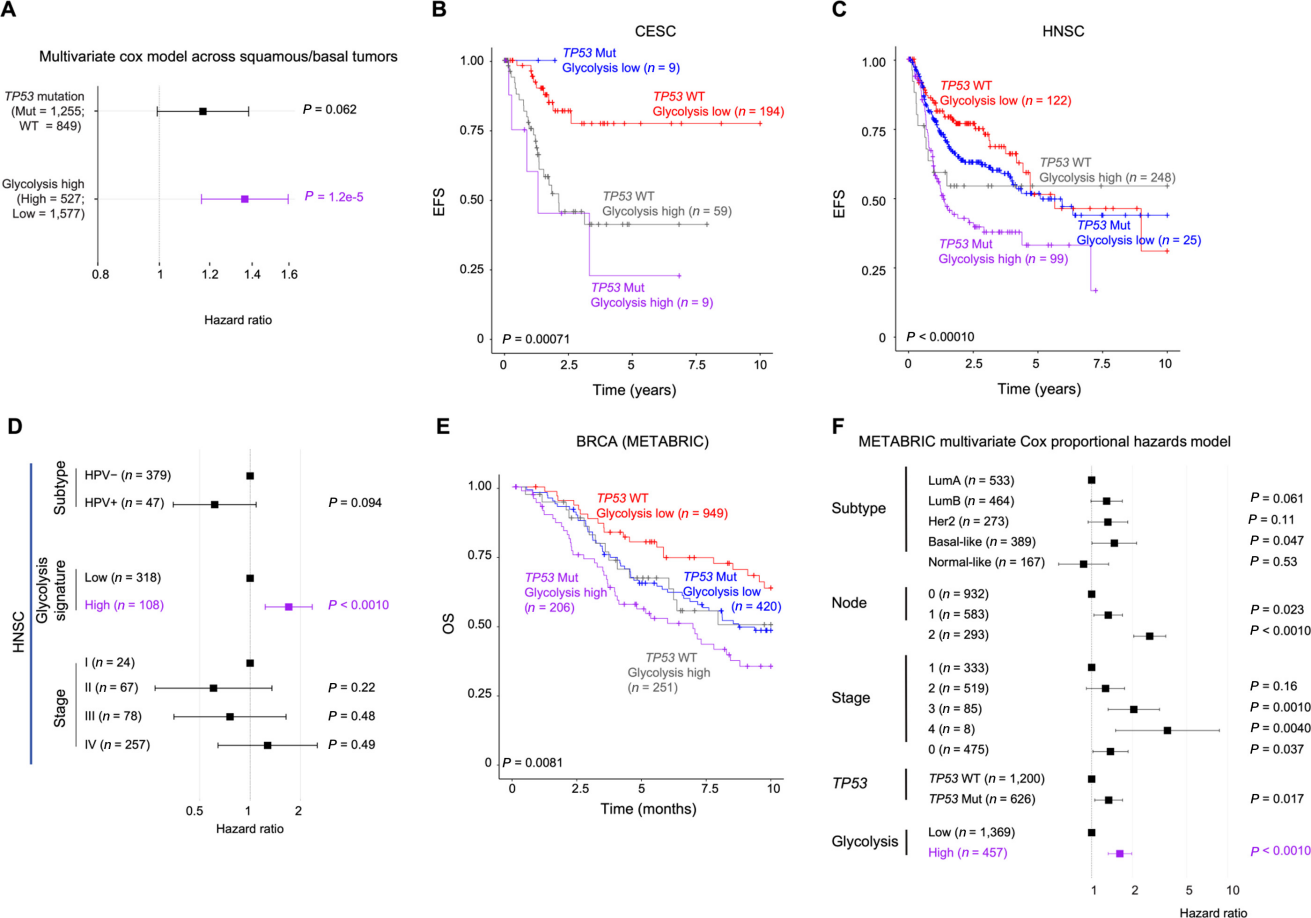
Elevated glycolysis signaling portends a worsened prognosis across squamous and breast cancers beyond clinical variables. **A,** Multivariate Cox proportional hazards model evaluating the impact of TP53 mutation and glycolysis signature [high (>third quartile) vs. low (<3rd quartile)] on EFS in 1,442 samples from 11 basal-like/squamous tumors excluding basal-like BRCA. Tumor subtype was employed as a covariate. Boxes and lines represent HRs and 95% CI, respectively. EFS of 271 CESC (**B**) and 494 HNSC (**C**) samples, stratified by TP53 mutation and glycolysis signaling. *P* value reported from log-rank test. **D,** Multivariate Cox proportional hazards model evaluating the impact of glycolysis signature and clinical factors on EFS in TCGA HNSC samples. Only features with univariate Cox model *P* value <0.20 were selected in the multivariate model. Boxes and lines indicate HRs and 95% CI, respectively. **E,** OS of 1,826 BRCA samples from METABRIC dataset, stratified by TP53 mutation and glycolysis signaling. **F,** Multivariate analysis within METABRIC breast cancer dataset. Only features with univariate Cox model *P* value <0.10 were selected in multivariate model. Boxes and lines indicate HRs and 95% CI, respectively. Features tested include: TP53 status, glycolysis signaling, clinical stage, pathologic grade, ER status (BRCA only), subtype, gender, and race (TCGA data only).

Clinical variables including TP53 status, glycolysis signaling, clinical stage, pathologic grade, nodal status, and subtype were analyzed by univariate analysis by coxph generating likelihood ratio *P* values using the survival package. For those clinical variables with univariate *P* values <0.1 were then considered in a multi-variate model. Forest plot for multi-variate Cox proportional model was displayed with ggforest from the survminer package (Fig. 6F).

### Survival Analysis

Clinical data from TCGA downloaded from the PanCan Atlas publication: clinical_PANCAN_patient_with_followup.tsv. From the 11,160 samples annotated, clinical data were narrowed to data with mutation and RNA-seq data and both event-free survival (EFS) and OS values present (*n* = 8,413).

Aneuploidy is defined as high if >15 and low ≤15. Glycolysis signature from ssGSEA Hallmark call was converted to binary score by first calculating the quartiles for each tumor type and next defining high glycolysis as tumors with ≥ third quartile. Survival data were censored at 10 years. Survival plots comparing *TP53* mutation and glycolysis score were analyzed by log-rank test and displayed by Kaplan–Meier curve from *survival* package using *ggsurvplot* (Fig. 6B and 6C).

Clinical variables including TP53 status, glycolysis signaling, clinical stage, pathologic grade, nodal status, gender, race, aneuploidy score, and subtype within each tumor type were analyzed by univariate analysis by coxph generating likelihood ratio *P* values using the survival package. For those clinical variables with univariate *P* values <0.2 were then considered in a multi-variate model. Forest plot for multi-variate Cox proportional model was displayed with ggforest from the survminer package (Fig. 6D).

### Other Statistical Analyses

Data visualization was performed using R v.3.6.2 within RStudio v.1.5033. Statistics and relevant information including the type and the number of replicates (*n*), the adopted statistical tests, and *P* values are reported in the figures and associated legends. Correlation tests were performed using cor.test function with method = “pearson,” and *P* values were corrected with p.adjust package method = “BH” to adjust for multiple hypothesis testing. Enrichment tests were performed with Fisher exact test within the mouse models and χ^2^ test for pan-cancer analyses, with OR and BH-adjusted *P* values reported.

### Data Access

The findings of this study are supported by data that are available from public online repositories and data that are publicly available upon request of the data provider. See Materials and Methods for detail.

## Results

### Pan-cancer Landscape of Hallmark Signatures

We first characterized gene expression of the 50 hallmark signatures from MSigDB (4) using ssGSEA 2.0 (28) called from RNA-seq data of 8,603 samples from TCGA (Supplementary Figs. S1 and S2A; Supplementary Table S1). Two signatures were excluded for low gene expression with little variability across the dataset. To elucidate the relationship among the hallmark signatures, we calculated samplewise comparisons across all signatures and compared the gene overlap of signatures. This analysis demonstrated many positive correlations across multiple signatures (even with low expression and/or low variability), despite having few gene overlap between them other than IFNα and IFNγ response signatures and the early and late estrogen response signatures (Supplementary Fig. S2B; Supplementary Table S2). To categorize highly correlated signatures, we then hierarchically clustered all signature scores across TCGA samples. This revealed eight significant groups of well-correlated signatures: lipid metabolism, proliferation, inflammation, apoptotic regulation, angiogenesis/epithelial-to-mesenchymal transition (EMT), developmental signaling, TGFβ signaling, and estrogen signaling (Supplementary Fig. S2C). Beyond the eight defined signature groups, positive correlations existed among the inflammation, apoptotic regulation, and angiogenesis/EMT signature groups (Supplementary Fig. S2B). Other correlations across signature groups included several unexpected relationships, such as oxidative phosphorylation with DNA repair and the p53 pathway with estrogen receptor signaling. Conversely, despite being a metabolic pathway, glycolysis was not correlated or even anticorrelated with most of the signatures in lipid metabolism (Supplementary Table S2).

Next, we evaluated the heterogeneity of hallmark signatures across 86 TCGA subtypes (with >10 samples and included in tumor types with >100 samples). Using a mean score of hallmark signatures for each subtype, hierarchical clustering revealed that most subtypes within a given tumor type grouped into the same cluster though with some notable exceptions (Fig. 1A).

Each cluster was then compared with all others to define cluster-specific signatures (Fig. 1B). Subtypes were first divided into two main groups by the proliferation signatures, dominated by the E2F targets and G_2_–M checkpoint signatures. Within the low proliferation group, three clusters were defined by low inflammation, high inflammation, or high lipid metabolism signaling. The low inflammation cluster included PRAD and the neuroendocrine/glial tumors [lower grade glioma (LGG) and PCPG], the latter of which exhibited increased developmental signaling. In contrast, the high inflammation cluster, including PAAD and KIRC, showed enhanced apoptotic and angiogenesis/EMT signatures. The high lipid metabolism cluster was dominated by LIHC and KIRP.

The high proliferation group was further classified into five clusters with significantly different signature patterns. The first cluster, defined by high inflammation and angiogenesis/EMT signatures, was comprised of squamous cell carcinomas and basal-like BRCA (Fig. 1C). Interestingly, basal-like BRCA was classified into this squamous cluster, apart from the other BRCA subtypes (Fig. 1D), reflecting distinct molecular and clinical features of the basal-like subtype from other BRCA subtypes (45). Within this cluster, human papillomavirus positive (HPV+) HNSC clustered more closely to squamous subtype of CESC, reflecting the underlying oncogenic virus driving these tumors, while HPV− HNSC clustered with other squamous tumors. One high-proliferative cluster characterized by high EMT signature consisted of mesenchymal tumors, including the sarcomas, mesenchymal subtype of OV, and isocitrate dehydrogenase (IDH) wild-type glioblastoma multiforme, of which more than one-third were previously reported to show a mesenchymal phenotype (ref. 46; Fig. 1E). High estrogen receptor signaling separated hormone receptor (HR)-driven BRCAs from all other tumors (Fig. 1D), while high inflammation characterized tumors with known immunologic involvement, including SKCM, Epstein–Barr virus positive STES, immunoreactive OV, THYM, and acute myeloid leukemia (LAML; Fig. 1F). These findings suggest that hallmark signatures successfully capture characteristic behaviors and phenotypes of tumors and unveil the similarities across subtypes regardless of the cell of origin.

### Effect of Somatic Mutations on Hallmark Signatures

Although a significant proportion of variation across the hallmark signatures can be explained by tumor type, many tumor types exhibit substantial heterogeneity. As genetic alterations can account for such intratumor type heterogeneity, next we evaluated the effect of somatic mutations on hallmark signatures. First, we narrowed the list of oncogenes to those with known hotspot mutations (30), and tumor suppressor genes (TSG; ref. 47) and defined significantly mutated genes within each tumor type and subtype (Supplementary Figs. S1C and S3A). This approach generated a list of 22 oncogenes and 37 TSGs, of which 10 oncogenes and 18 TSGs were mutated in at least two tumor types (for a total of 120 gene–tumor type combinations; Supplementary Table S4; Supplementary Fig. S1D and S1E). Next, to detect robust relationships between signatures and mutations in multiple tumor types, we investigated the associations significantly consistent across tumor types using permutation testing (Supplementary Tables S5 and S6; Supplementary Fig. S1C–S1F). Analyzing within each tumor type, 376 significant signature–mutation relationships were identified in at least one tumor type (546 signature–mutation–tumor type combinations in total) were identified; however, with permutation testing, only 65 signature–mutation relationships (219 mutation–signature–tumor type combinations in total) were consistently significant across tumor types (Fig. 2A; Supplementary Table S7). These included well-described relationships, such as *CTNNB1* mutation with WNT/β-catenin signaling, *NFE2L2* mutations with reactive oxygen species (ROS), *RB1* mutation with E2F targets, and *TP53* mutation with proliferation-related signatures, confirming the validity of our method (Supplementary Fig. S3B).

Among these relationships, we also identified many significant relationships which have not been reported in a large cohort of clinical samples. A considerable proportion of these relationships consisted of *TP53* mutation with multiple signatures, including upregulation of glycolysis and MTORC1 signaling and down-regulation of lipid metabolism signatures (Fig. 2B). According to the recent large-scale functional assessment, we classified *TP53* missense mutations further into impactful I, impactful II, and not otherwise classified (48), demonstrating similar activation of glycolysis across the missense and nonsense mutations (Supplementary Fig. S3C). These results suggest that *TP53* mutations induce a wide array of hallmark signature disruption that cannot be explained by the direct effect of p53 transcriptional regulation.

*CDH1* mutation affected the second broadest range of signatures, demonstrating the opposite effect of *TP53* mutation in 10 signatures, including the downregulation of proliferation signatures as well as glycolysis and MTORC1 signaling (Fig. 2B). Consistent with these findings, metabolomic data from CCLE demonstrated that *TP53*-mutated cell lines had upregulated glycolysis-related metabolites (Fig. 2C), while *CDH1* mutation caused their downregulation (Fig. 2D). Other significant relationships included *BRAF* mutation with upregulated angiogenesis and EMT signaling and downregulated PI3K-AKT-MTOR signaling and *PTEN* mutation and upregulated glycolysis, angiogenesis, and coagulation (Fig. 2A; Supplementary Fig. S3D). Although a variety of hallmark signature changes were observed in subtypes defined by *BRAF* mutation in SKCM and THCA (Fig. 1A), only a small portion were consistently observed across these two tumor types (Supplementary Fig. S3E).

Given that selected mutations are known subtype-defining events, relationships between signatures and mutations can be partly explained by tumor subtypes. For example, among the BRCA subtypes, glycolysis and MTORC1 signature scores were in proportion to the frequency of *TP53* mutation and inversely correlated with that of *CDH1* mutation (Fig. 2E); however, even within specific subtypes including LumA BRCA, these relationships were significant (Fig. 2F; Supplementary Fig. S3F). Among the significant relationships identified by our permutation framework, 61% of them were still significant when adjusted for subtype (Supplementary Fig. S3G; Supplementary Table S8). Therefore, although mutation-associated hallmark signature changes can be due to characteristic features of the heterogeneity across tumor subtypes, the majority of the identified relationships affect signatures beyond and within a subtype.

### Similar Effects on Hallmark Signatures Between TP53 Mutation and Arm-level CNAs

Utilizing arm-level CNAs filtered for significance within each tumor type and subtype by GISTIC2.0 (31), we next examined the associations of 29 arm-level amplifications and 29 deletions observed in at least two tumor types (471 arm-level CNA–tumor type combinations) with hallmark signatures (Supplementary Tables S5 and S6). Although 401 signature – arm-level CNA relationships were considered significant in each tumor type analysis, our permutation framework identified only 73 relationships consistently significant across multiple tumor types (Fig. 3A; Supplementary Table S7). Even when adjusting for subtype, 63% of the relationships were still significant (Supplementary Fig. S4A; Supplementary Table S8). Among them, well-established relationships, such as 17p deletion (containing *TP53*) with E2F targets and G_2_–M checkpoint, 10q deletion (containing *PTEN*) with angiogenesis, and 8q amplification (containing *MYC*) with MYC signaling were present, also confirming the reliability of our approach (Fig. 3A).

Most arm-level CNAs associated with hallmark signatures were deletions. Apart from inflammation signatures, these arm-level CNAs resembled changes of hallmark signatures by *TP53* mutation with an increase in proliferation and glycolysis and reciprocal downregulation of lipid metabolism and p53 signaling (Fig. 3A). These changes were also consistent with WGD, the process by which additional copies of the entire genome are generated (Fig. 3A). This signature pattern was increasingly significant when all arm-level CNAs were evaluated together as an aneuploidy score, consistent with prior literature (8). 5q deletion was associated with the widest range of signature changes, including glycolysis and inflammation signatures (Fig. 3A). These relationships were evident even within specific tumor subtypes, such as the chromosomal instability (CIN) subtype of STES and HPV− HNSC (Supplementary Fig. S4B). Notably, although *TP53* mutation is known to be associated with increased aneuploidy, even within *TP53* wild-type tumors, significant relationships reflected the analysis in the entire cohort especially with arm-level deletions and increased proliferation signatures (Supplementary Fig. S4C). Taken together, both genomic instability overall and specific arm-level CNAs drive similar changes in hallmark signatures with *TP53* mutation.

### Focal CNAs Show Aneuploidy-like and Gene-specific Patterns in Relationships with Hallmark Signatures

Next, we analyzed the relationship between focal CNAs and hallmark signatures. By calling focal CNAs with GISTIC 2.0 (31) within each tumor type and subtype, we identified 28 focal amplifications and 33 focal deletions significantly altered for a total of 226 focal CNA–tumor type combinations and collapsed these significant focal CNAs to the gene level (Supplementary Fig. S5A). We found 462 significant signature–focal CNA–tumor type relationships representing 182 consistent focal CNA–signature relationships (Fig. 3B and C; Supplementary Table S6). Among them, 43% retained significance even after adjusting for subtype (Supplementary Fig. S5B; Supplementary Table S8), suggesting that half of these relationships can be associated with the previously defined subtypes but the other half are novel relationships beyond subtype. These included well-described relationships, such as *RB1* deletion with E2F targets, *MYC* amplification with MYC targets, and *PTEN* deletion with angiogenesis. Like arm-level CNAs, a substantial proportion of focal CNAs showed a similar relationship pattern to the *TP53* mutation and aneuploidy score. Many of these focal CNAs co-occurred with arm-level alterations (Supplementary Fig. S5C and S5D), reflecting the consistency between focal and arm-level CNAs.

Apart from *TP53* mutation/aneuploidy-related signature changes, consistent relationships were observed between angiogenesis and EMT and focal CNAs across multiple tumor types (Fig. 3B and C). Specifically, *CDKN2A* and *PTEN* deletions were associated with upregulation of angiogenesis, while *CREBBP* deletion was associated with its downregulation. In addition, *CDKN2A* deletion and *CDK4* amplification were related to EMT upregulation. Other notable relationships included *RB1* deletion with decreased NOTCH signaling, *CDKN2A* and *FAT1* deletions with increased glycolysis, and *CDKN2A* deletion with reduced inflammation signatures. Even within subtypes, *CDKN2A* deletion leads to EMT upregulation within IDH wild-type LGG, HPV + HNSC, and 4-KIRC (Supplementary Fig. S5E). Within IDH wild-type LGG, *CDKN2A* deletion and *PTEN* deletion upregulate angiogenesis signaling (Supplementary Fig. S5F and S5G). Although many relationships between signatures and driver genes have been implicated by biological experiments within a given tumor type (4, 49), our analyses extend these significant relationships across tumor types in a large-scale patient dataset.

### TP53 Mutation is Associated with a Specific Spectrum of CNAs in the Basal-like/Squamous Cluster

*TP53* mutation is known to be associated with increased aneuploidy, reflected in the similar signature changes across the *TP53* mutation and CNA analyses; however, whether *TP53* mutation is associated with widespread genomic instability or generates a specific spectrum of CNAs is not well understood. Therefore, we next investigated the landscape of CNAs across tumor subtypes and their association with *TP53* mutation.

Hierarchical clustering of the mean arm-level CNAs in a given subtype demonstrated some intratumor type heterogeneity across tumor subtypes, similar to the initial hallmark signature cluster (Fig. 4A). Among 12 identified clusters, the most prominent was a cluster containing tumor subtypes with frequent *TP53* mutation and high aneuploidy scores (Fig. 4B). This cluster consisted not only of squamous cell carcinomas but also of OV, basal-like BRCA, and basal/squamous-like BLCA. Apart from basal-like BRCA, the HR-driven BRCAs were significantly classified into a separate cluster with a moderate frequency of *TP53* mutation, containing LUAD, PAAD, and LIHC, consistent with the previous PanCan CNA clustering results (ref. 8; Fig. 4C). The basal-like/squamous cluster demonstrated a significantly high degree of overlap with the squamous cluster identified from the hallmark signatures, including HNSC, CESC, LUSC, esophageal squamous cell carcinoma (ESCC) STES, basal/squamous-like BLCA, and basal-like BRCA (Fig. 4D).

As the above CNA squamous subtype cluster showed a characteristic pattern of both arm-level CNAs and a high percentage of *TP53* mutation, we then compared arm-level CNAs between *TP53* wild-type and mutated samples. 5q deletion and 17p deletion were the most highly recurrent arm-level CNAs in *TP53*-mutated tumors (Fig. 4E; Supplementary Table S9). Compared with the other tumor subtypes, 5q deletion was also significantly enriched within the identified squamous cluster, where reciprocal 5p amplification co-occurred in most samples (Supplementary Fig. S6A–S6C). Even within tumor subtypes of this cluster, 5q deletion was more common in *TP53*-mutated samples, with the highest percentages in basal-like BRCA and LUSC (Supplementary Fig. S6D). We next determined the chronological order of CNAs significantly enriched in *TP53*-mutated tumors relative to *TP53* mutation, using WGS data from basal-like BRCA. Many deletions tended to occur after *TP53* mutation but before WGD, while amplifications arose after *TP53* mutation, irrespective of the WGD timing (Fig. 4F). These observations suggest that, although *TP53* mutation is associated with widespread genomic instability, a specific spectrum of arm-level CNAs is clonally selected in *TP53* mutated tumors.

### TP53 Mutation Cooperates with Aneuploidy to Induce Enhanced Glycolysis in the Basal-like/Squamous Tumor Types

Given the selection of these wide-spread CNAs following *TP53* mutation, we hypothesized that general aneuploidy and specific CNAs such as 5q deletion further augment changes in hallmark signature within *TP53*-mutated tumors. Although aneuploidy exerted diverse hallmark signature changes similarly to *TP53* mutation when analyzed in all tumors (Fig. 3A), within the *TP53*-mutated basal-like/squamous tumors, aneuploidy had an effect beyond *TP53* mutation (Fig. 5A). Among them, the most significant upregulated signature was glycolysis. The cooperation of *TP53* mutation and aneuploidy to drive enhanced glycolysis was evident within the squamous cluster tumor types (Fig. 5B. To a lesser extent, 5q deletion also augmented *TP53* mutation to upregulate glycolysis signature within the squamous tumor types (Fig. 5C; Supplementary Table S10). These findings suggest that aneuploidy and specifically selected arm-level CNAs augment *TP53* mutation to enhance the malignant phenotype, such as upregulated proliferation and glycolysis, in these basal-like/squamous tumors.

### Breast Cancer Mouse Models Recapitulate the Coordinated Effect of TP53 Mutation and Both Aneuploidy and Specific CNAs

To further investigate the relationship among *TP53* mutation, CNAs, and hallmark signatures, we utilized GEMMs of breast cancers containing either wild-type or germline *Trp53* deletion (Supplementary Table S11; refs. 38–40, 41). First, we examined microarray expression data of these tumors, validating our findings from the human data that indeed *Trp53*-null tumors have increased proliferation and glycolysis signaling compared with *Trp53* wild-type tumors (Fig. 5D). We next defined secondary CNA events within *Trp53* wild-type versus null GEMMs to identify the effect of *Trp53* germline mutation on genomic instability. *Trp53*-null mouse tumors had increased aneuploidy compared with *Trp53* wild-type tumors (Fig. 5E). Frequency plot of CNAs demonstrated a consistent pattern of arm-level deletion and amplification between two different *Trp53* models (*Trp53-*null BALBc model and the *Trp53-*null/*Brca1-*null FVB model KPB1B; Supplementary Fig. S7), including deletion of the mouse homolog to human 5q. Aneuploidy further augmented glycolysis signaling within the *Trp53-*null tumors (Fig. 5F). These data further support the notion that specific CNAs in a background of widespread genomic instability act as second hits to *Trp53* mutation, augmenting *Trp53* loss to drive a more aggressive phenotype observed in both basal-like BRCA and the squamous tumors.

### Prognostic Impact of Upregulated Glycolysis Signaling within Squamous Tumors

As enhanced glycolysis signaling is a shared consequence of *TP53* mutation and resultant genomic instability in squamous tumors, we next examined the effect of glycolysis signaling on both EFS and OS using TCGA data (*n* = 1,583 for the 12 squamous cluster tumor subtypes). Strikingly, a multivariate analysis incorporating glycolysis signature, *TP53* status, and subtype demonstrated that high glycolysis signaling significantly predicts worse survival outcomes, independent of *TP53* mutation (Fig. 6A; Supplementary Fig. S8A). Particularly, elevated glycolysis signaling worsened EFS beyond *TP53* mutation in both CESC and HNSC (Fig. 6B and C). Furthermore, in a multivariate model including glycolysis signature, *TP53* status, subtype, and several clinical factors, high glycolysis independently predicted worsened EFS within both HNSC and CESC (Fig. 6D; Supplementary Fig. S8A).

Because of insufficient follow-up time within TCGA BRCA cohort (45), we employed an independent dataset of breast cancers, the METABRIC (refs. 43, 44; *n* = 1,826), a large, highly curated publicly available breast cancer dataset with genomic, transcriptomic, and clinical data. Within METABRIC, the relationships across hallmark signatures, mutations, arm-level CNAs, and focal CNAs were extremely consistent with TCGA BRCA analyses (Supplementary Fig. S8B–S8E). METABRIC BRCA samples with *TP53* mutation and elevated glycolysis signaling had the worst OS (Fig. 6E; Supplementary Table S12). Glycolysis signaling was significant even when adjusted for subtype, *TP53* mutation, and clinical features in a multivariate model (Fig. 6F). Taken together, across the basal-like and squamous tumor types/subtypes, high glycolysis signaling has independent prognostic value, predicting a worse clinical outcome even in the setting of *TP53* mutation and high degrees of aneuploidy.

## Discussion

Although hallmark signatures efficiently capture diverse cancer phenotypes, their heterogeneities across subtypes and the underlying somatic alterations driving them remain unclear. Therefore, in this work, we present a systematic analysis of hallmark signatures and their somatic drivers in human cancers, revealing many alterations explaining signature changes consistent across multiple tumor types. Among them, we show a consistent relationship of *TP53* mutation and specific CNAs with diverse hallmark signature changes. In pan-cancer hallmark signature and CNA analyses, we define a consistent cluster of squamous tumors which clusters the basal-like subtypes of both BRCA and BLCA together. This cluster is characterized by enhanced proliferation and inflammation signatures, frequent *TP53* mutation, and a characteristic pattern of aneuploidy including a high frequency of 5q deletion. Within the squamous cluster tumor subtypes, *TP53* mutation and aneuploidy cooperate to enhance high glycolysis signaling, which is demonstrated as an independent prognostic factor in patients with these subtypes.

In *TP53*-mutated tumors, 5q deletion is the most preferentially selected arm-level CNA. Interestingly, 5q deletion is also the most frequent arm-level CNA in *TP53*-mutated tumors with myelodysplastic syndrome (MDS; refs. 50, 51), suggesting 5q is a common target of deletion in *TP53*-mutated solid and hematologic cancers. In MDS, 5q deletion causes haploinsufficiency of several genes (52), including *RPS14*, *CSNK1A1*, *APC*, *DDX41*, and miR-145/miR-146a, leading to deregulated p53-mediated apoptosis, WNT/β-catenin, and inflammatory signaling. In addition to these genes, many 5q genes are involved in diverse hallmark signature changes driving oncogenesis: *RAD17* and *RAD50* are directly involved in DNA double-strand break repair (53); *MAP3K1* is a repressor of the mitogen-activated protein (MAP)-kinase pathway and regulates several survival and/or proliferation pathways (54); and *PLK2* is involved in MTORC1 signaling (55) and as a biomarker for treatment in triple-negative breast cancer (56). Further experimental work is required to explore the individual consequence of deletion of these genes within the 5q region in the basal-like/squamous tumors.

A significant contribution of this work is the identification of consistent molecular changes across basal-like BRCA, basal/squamous-like BLCA, and squamous tumors. While prior work has demonstrated consistent features such as 3p deletion between basal/squamous-like BLCA and squamous tumors (49), this is the first systematic demonstration of widespread genetic and transcriptomic similarities of basal-like BRCA to the squamous tumors. Furthermore, the consistent separation of basal-like BRCA from the other BRCA subtypes signifies the importance of considering subtype in genomic studies rather than a tumor type-only approach. Despite a lack of histologic similarity and thus the usage of the term “basal-like” for BRCA, the consistent molecular features of *TP53* mutation, 5q deletion, and hallmark signature changes of high proliferation and glycolysis signaling across these tumor types are clinically meaningful, with marked worsened prognosis in these tumors. Glycolysis not only helps management of cellular bioenergetics for maintaining uninterrupted growth, but also facilitates immune evasion and mitigates excessive accumulation of ROS by circumventing mitochondrial oxidative phosphorylation (57, 58). These roles may contribute to the progression of the basal-like/squamous tumors, which show high proliferation and high inflammation signatures. Potential therapeutic interventions targeting glycolysis could be applied across these tumor types, although further investigation for the causal relationship between TP53 mutation, aneuploidy and upregulated glycolysis is required.

There are several limitations in our study: both intrinsic and extrinsic properties can affect hallmark signatures. In addition, several somatic alterations are reported to have different functions when co-occurring with a specific alteration, such as a combination of *TP53* and *KRAS* mutations. Thus, further investigations, including those with discriminating microenvironmental cells using single-cell RNA-seq and taking a combination effect into account, are warranted. As we approach an era in which clinical sequencing becomes routine in cancer care, the genetic complexity of cancer continues to challenge the limits of using genomic alterations to predict prognosis and therapeutic response. By supervising our analysis by tumor subtypes, selecting a catalogue of robust somatic alterations, and identifying consistent dysregulation of cancer hallmark signatures, our study provides a unique approach to coalesce large genomic datasets, providing a unifying link between hallmark signatures of cancer and genetic alterations. In addition to the relationships with *TP53* mutations, we identified many significant relationships, such as *BRAF* and *PTEN* mutations with upregulated angiogenesis, which can be exploited to develop novel predictive and therapeutic strategies. Elucidation of these relationships leads to a better unified understanding of oncogenic mechanisms and the improvement of patient management across cancer.

## Supplementary Material

Supplementary Tables 1-12 Supplemental Table 1. Samples utilized from the Cancer Genome Atlas Project (TCGA) including tumor type, subtype assignment, and citation for subtype assignment.
Supplemental Table 2. Pearson correlation and Jaccard index comparing all 48 Hallmark Signatures in pairwise comparisons using the full 8,603 tumors from TCGA.
Supplemental Table 3. Summary of number in tumor types and subtypes.
Supplemental Table 4. Significant somatic alterations including somatic mutations, focal copy number alterations (CNAs), and arm-level CNAs for each tumor type and subtype. (TSG = tumor suppressor gene).
Supplemental Table 5. Number of significant tumor types per somatic alteration.
Supplemental Table 6. Results of Wilcoxon rank sum test of somatic alterations versus Hallmark signatures in tumor type analyses.
Supplemental Table 7. Permutation test result of somatic alterations versus Hallmark signatures.
Supplemental Table 8. Results of Wilcoxon rank sum test of somatic alterations versus Hallmark signatures in subtype analyses.
Supplemental Table 9. c2 testing of TP53 mutant versus wild-type tumors and squamous/basal tumors versus all other tumors for arm-level CNAs.
Supplemental Table 10. Linear mixed model of TP53 mutated tumors within the basal/squamous subtypes comparing hallmark signature score to aneuploidy score with tumor subtype as a covariate.
Supplemental Table 11. Genetically engineered mouse model sample information. (aCGH = competitive genomic hybridization array).
Supplemental Table 12. Wilcoxon rank sum test of somatic alterations versus hallmark signature scores utilizing data from the Molecular Taxonomy of Breast Cancer International Consortium.

Supplementary Figure Legends 1-8 Figure S1. Methods workflow for pan-cancer analysis of hallmark signatures.
Figure S2. Characteristics of hallmark signatures across TCGA and tumor subtypes.
Figure S3. Validation of mutation – hallmark signature relationships across TCGA.
Figure S4. Validation of arm-level alteration relationship with both hallmark signatures and TP53 mutation.
Figure S5. Identification of robust focal alterations and confounding results of mutations and arm-level alterations.
Figure S6. Overlap and 5q deletion of basal-like BRCA and squamous tumors compared to HR-driven BRCA.
Figure S7. Overview and detailed copy number aberrations of Trp53 wild-type and null genetically engineered mouse models of human breast cancer.
Figure S8. Analysis of mutations, hallmark signatures, and multivariate analysis of survival in a large independent primary breast cancer dataset.

Supplementary Figures 1-8 S1. Methods workflow for pan-cancer analysis of hallmark signatures.
S2. Characteristics of hallmark signatures across TCGA and tumor subtypes.
S3. Validation of mutation – hallmark signature relationships across TCGA.
S4. Validation of arm-level alteration relationship with both hallmark signatures and TP53 mutation.
S5. Identification of robust focal alterations and confounding results of mutations and arm-level alterations.
S6. Overlap and 5q deletion of basal-like BRCA and squamous tumors compared to HR-driven BRCA.
S7. Overview and detailed copy number aberrations of Trp53 wild-type and null genetically engineered mouse models of human breast cancer.
S8. Analysis of mutations, hallmark signatures, and multivariate analysis of survival in a large independent primary breast cancer dataset.
