# Supplementary material for: Landscape of Genetic Alterations Underlying Hallmark Signature Changes in Cancer Reveals TP53 Aneuploidy–driven Metabolic Reprogramming
Source: Cancer Res Commun. 2023 Feb 16;3(2):281–96. doi: 10.1158/2767-9764.CRC-22-0073 (PMC9973382; doi:10.1158/2767-9764.CRC-22-0073)
Supplement: Supplementary Figure Legends 1-8 — Figure S1. Methods workflow for pan-cancer analysis of hallmark signatures. Figure S2. Characteristics of hallmark signatures across TCGA and tumor subtypes. Figure S3. Validation of mutation – hallmark signature relationships across TCGA. Figure S4. Validation of arm-level alteration relationship with both hallmark signatures and TP53 mutation. Figure S5. Identification of robust focal alterations and confounding results of mutations and arm-level alterations. Figure S6. Overlap and 5q deletion of basal-like BRCA and squamous tumors compared to HR-driven BRCA. Figure S7. Overview and detailed copy number aberrations of Trp53 wild-type and null genetically engineered mouse models of human breast cancer. Figure S8. Analysis of mutations, hallmark signatures, and multivariate analysis of survival in a large independent primary breast cancer dataset. [file crc-22-0073-s02.docx]

# Supplemental Figure Legends

### Supplemental Figure S1. Methods workflow for pan-cancer analysis of hallmark signatures.

(A) Hallmark signature scores from 10,344 tumors were calculated using ssGSEA 2.0(14) and the MSigDB Hallmark dataset(4). (B) Signature expression from the 8,603 samples with both RNA-seq and mutation data were evaluated with interquartile range, and two signatures removed due to low expression. (C) To robustly identify somatic alterations taking into account the variety in mutation rate and overall genomic instability by tumor type and subtype, we first narrowed mutations, focal alterations, and arm-level alterations to those significantly enriched within each tumor type or tumor subtype. For 37 TSGs, we calculated the statistical outlier for each tumor type and each tumor subtype: of the number of tumors with a specific gene mutated, those genes above the 3^rd^ quartile + 3 × interquartile range (IQR), had less than 20% synonymous mutations, and at least 3 mutations in the dataset. For 28 oncogenes, only hotspots published in Hess et al. 2019(17) were considered, and a similar cutoff defined as 3^rd^ quartile + 3×IQR and at least 3 mutations were maintained. Significant hits were then collapsed to the tumor type level and maintained for down-stream analyses. For focal copy number alterations (CNAs), ISAR segmented PanCan data was input into GISTIC 2.0(20) for each tumor subtype and each tumor type considering a segment cutoff of 0.10 and arm-level alteration at >50% of the arm altered. Segments with a q-value <0.01 and width of <250,000 bases were collapsed to the gene level, and the union within a tumor type if multiple subtypes were present was considered. TCGA GISTIC ISAR gene-level copy number calls from Hoadley et al. were utilized for downstream analyses. Samples with both RNA-seq and mutation data were input into Wilcoxon rank sum test and permutation testing (n = 8,264). For arm-level CNAs, ISAR segmented PanCan data input into GISTIC 2.0 for each tumor subtype and tumor type produced significant arm-level alterations (q value <1.0×10^-3^). Next, binary arm-level calls from Taylor et al.(8) were filtered to those arm-level alterations defined in GISTIC analysis (71 arm-level CNAs) and utilized for downstream analyses. Samples with both RNA-seq and mutation data were input into Wilcoxon rank sum test and permutation testing (n = 8,231). (D) Summary of significantly altered somatic changes. (E) Summary of somatic alterations significant in at least two tumor types which were then input into permutation testing: 28 mutations from 2-21 tumor types, 61 focal CNAs from 2-19 tumor types, and 58 arm-level CNAs from 2-22 tumor types. Samples were tested if they had both RNA-seq and mutation data (8,603 for mutations, 8,264 for focal CNAs, and 8,231 for arm-level CNAs). (F) Permutation testing to establish false positive rate for each signature – somatic alteration across 1,000,000 permutations. (G) Significant signature – somatic alteration relationships with BH-adjusted FDR for mutations (q ≤1.0x10^-4^), focal CNAs (q ≤1.0x10^-4^), and arm-level CNAs (q ≤1.0x10^-7^).

### Supplemental Figure S2. Characteristics of hallmark signatures across TCGA and tumor subtypes.

(A) Interquartile range per hallmark signature for 8,603 samples was calculated within each tumor subtype and plotted across hallmark signatures to evaluate overall expression and distribution of scores. Horizontal line marks median signature score. (B) Cross-signature correlation and Jaccard index among each of the 48 hallmark signatures compared to all others. Signatures are colored by highly significant positive relationships as determined by Pearson correlation across all of TCGA (n = 8,603). Only positive signature-signature relationships with BH-adjusted p-value <1.0×10^-50^ are shown. (C) ssGSEA hallmark signature scores from the TCGA cohort (n = 8,603), median centered, and hierarchically clustered with Pearson correlation and Ward linkage method. Red = up-regulation; blue = down-regulation of signature. Side bar indicates tumor type and tumor subtype as defined in Fig. 1.

### Supplemental Figure S3. Validation of mutation – hallmark signature relationships across TCGA.

(A) Distribution of tumor suppressor mutations within each tumor subtype. Within each tumor subtype, a cutoff is defined by first counting the number of tumors with non-synonymous mutations, identifying the 3^rd^ quartile + 3×IQR, and filtering mutations to genes (1) at or above this threshold and (2) with <20% silent mutations. Genes included in downstream analysis (pink) and excluded mutations (blue). (B) Kernel density plot of known relationships between up-regulation of reactive oxygen species signaling with mutations in *NFE2L2* across LUSC and STES. (C) Kernel density plot of glycolysis comparing wild-type, and *TP53* missense and nonsense mutations across BRCA, HNSC, LUAD, and STES. Missense mutations are further categorized into Impactful I, Impactful II, and NOS (19). Wilcoxon rank sum p-values are displayed for each wild-type – mutation comparison. (D) Kernel density plots of angiogenesis, apoptosis, and glycolysis comparing *PTEN* mutation in both LGG and UCEC tumor types. Wilcoxon rank sum p-values are displayed for each signature – mutation combination. (E) Effect size of *BRAF* mutation across SKCM and THCA for significant hallmark signatures. Error bars display the 95% confidence interval from Wilcoxon rank sum test. (F) Kernel density plots of G2M checkpoint, glycolysis, and MTORC1 signaling for subtypes across both BRCA and STES comparing *CDH1* wild-type (black) versus mutant (green) tumors. (G) Percent of significant signature – mutation relationships in linear mixed modeling with subtype as covariate. BH-adjusted p-values <0.050 were considered as significant. Purple = signature – mutation relationship significant beyond subtype.

### Supplemental Figure S4. Validation of arm-level alteration relationship with both hallmark signatures and TP53 mutation.

(A) Percent of significant signature – arm-level CNA relationships in linear mixed modeling with subtype as covariate. BH-adjusted p-values <0.05 were considered as significant. Purple = signature – arm-level CNA relationship significant beyond subtype. (B) Kernel density plot of G2M checkpoint signature score within tumor subtypes in 5q neutral versus deleted tumors for both tumor type level analysis in BRCA, LUSC, UCEC, and STES as well as subtype analysis for both BRCA and STES subtypes. Horizontal bar indicates median for each category. Wilcoxon rank sum p-values were displayed. (C) Of those signatures significant in arm-level CNA – hallmark analysis, Wilcoxon rank sum test re-performed within *TP53* wild-type tumors. Color and dot size indicate fold-change (red = up-regulation; blue = down-regulation) and number of significant tumor types, respectively.

### Supplemental Figure S5. Identification of robust focal alterations and confounding results of mutations and arm-level alterations.

(A) GISTIC output of focal alterations filtered to q <0.01 and width of 250,000 bases and collapsed to gene level, displayed across the genome with the closest cancer-related genes labeled. Red = focal amplification; blue = focal deletion. (B) Percent of significant focal CNA – signature relationships when performing linear mixed modeling with subtype as covariate. BH-adjusted p-values <0.05 were considered as significant. Purple = signature – focal CNA relationship significant beyond subtype. (C) For each focal alteration significant within a tumor type, the percent of tumors within that tumor type that have either the focal alteration and arm-level alteration in the same direction (purple) or no/opposite arm-level alteration (gray). *DDAH1* amplification*, MECOM* amplification, *CDKN2A* deletion, and *RB1* deletion for BRCA, LIHC, and LUAD are displayed. (D) Box and whisker plot displaying the median, upper, and lower quartiles for each focal CNA – tumor type (Amp = 96 from 28 genes; Del = 128 from 33 genes) with or without concurrent arm-level alteration occurring in the same direction. Dots colored by gene tested.

### Supplemental Figure S6. Overlap and 5q deletion of basal-like BRCA and squamous tumors compared to HR-driven BRCA.

(A) SWITCH plot showing the frequency of copy number alteration generated from segmentation data of tumors within the 12 basal-like/squamous subtypes (n = 1,664), ordered by genomic location. Red = amplification; blue = deletion. (B) χ^2^ test of basal-like/squamous tumors (n = 1,664) versus all other tumors for each copy number alteration. Odds ratio of arm-level alteration compared within the squamous cluster (x axis) versus the BH-adjusted p-value (y axis). Red = amplification; Blue = deletion. (C) Segmentation for each basal-like/squamous tumor across chromosome 5. Each row represents one tumor, red = segment ratio ≥0.50; blue = segment ratio ≤ -0.50. (D) Percent of tumors within each subtype with 5q deletion. Gray = *TP53* wild-type tumors, blue = *TP53* mutated tumors. Two-sided t-test comparing *TP53* wild-type versus mutant tumors for each subtype.

### Supplemental Fig. S7. Overview and detailed copy number aberrations of *Trp53* wild-type and null genetically engineered mouse models of human breast cancer.

Frequency of copy number alteration for *Trp53* wild-type tumors, *Trp53-*null, luminal BALB/c tumors, *Trp53-*null basal-like BALB/c tumors, and KPB1B FVB tumors.

### Supplemental Figure S8. Analysis of mutations, hallmark signatures, and multivariate analysis of survival in a large independent primary breast cancer dataset.

(A) Hazard ratio (HR) of glycolysis signaling in high (>3^rd^ quartile) versus low (≤ 3^rd^ quartile) tumors for the basal-like/squamous tumor types and subtypes for TCGA and METABRIC. Boxes and lines indicate HR and 95% confidence interval, respectively. Color is proportionate to the -log10 BH-adjusted p-value for each comparison. Circles indicate overall survival while squares indicate event free survival. (B) t-statistic of mutations within BRCA TCGA (n = 1,074) and METABRIC (n = 1,826) for each signature – mutation relationship. T-statistic for each C-E, (C) signature – mutations, (D) signature – focal CNAs, and (E) signature – arm-level CNAs significant within the BRCA TCGA dataset in TCGA (x axis) and METABRIC (y axis). Significance (green) is defined as a Wilcoxon rank sum p-value ≤1.0x10^-3^.
