# Supplementary figures and images for: Landscape of Genetic Alterations Underlying Hallmark Signature Changes in Cancer Reveals TP53 Aneuploidy–driven Metabolic Reprogramming
Source: Cancer Res Commun. 2023 Feb 16;3(2):281–96. doi: 10.1158/2767-9764.CRC-22-0073 (PMC9973382; doi:10.1158/2767-9764.CRC-22-0073)

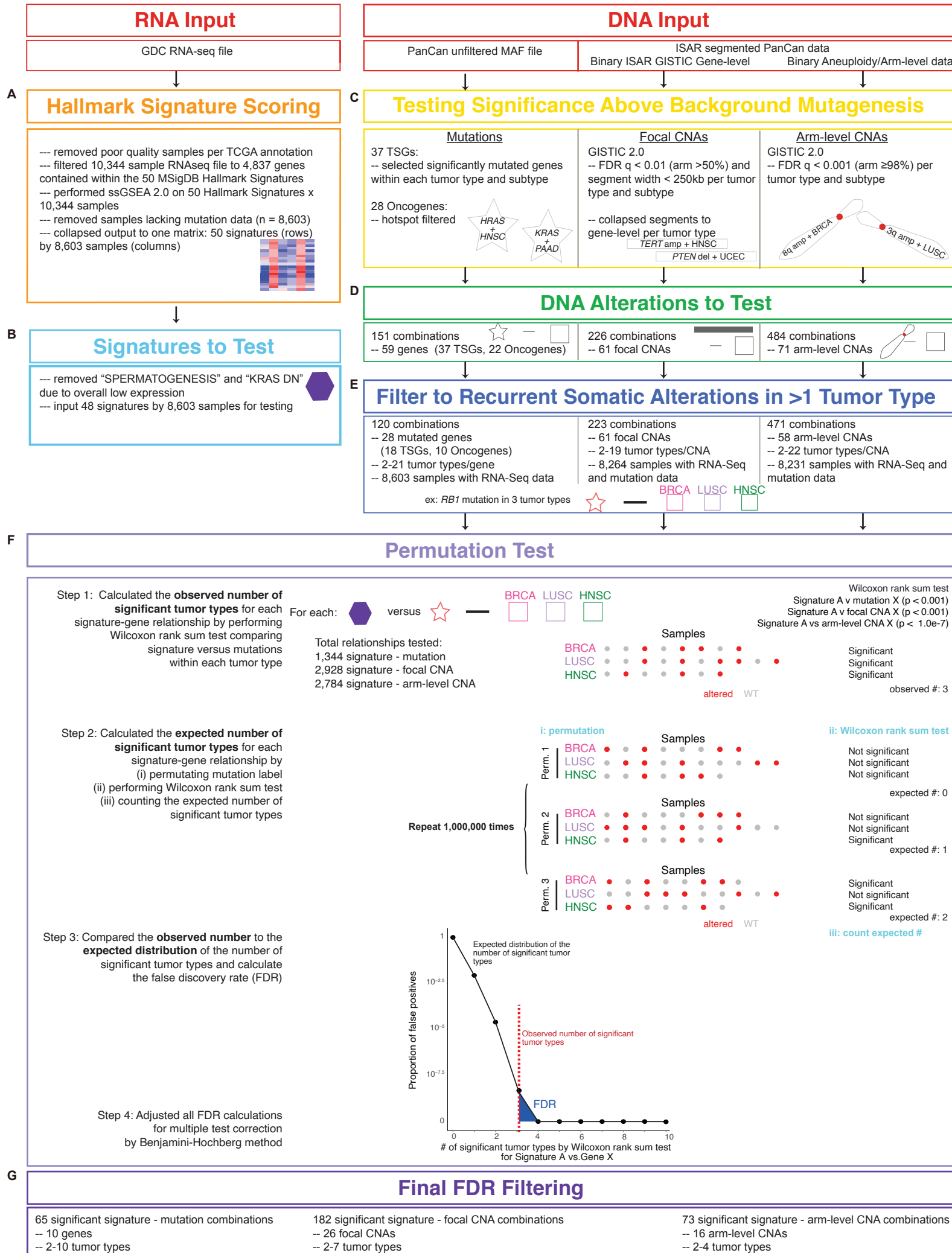

Figure S1

A

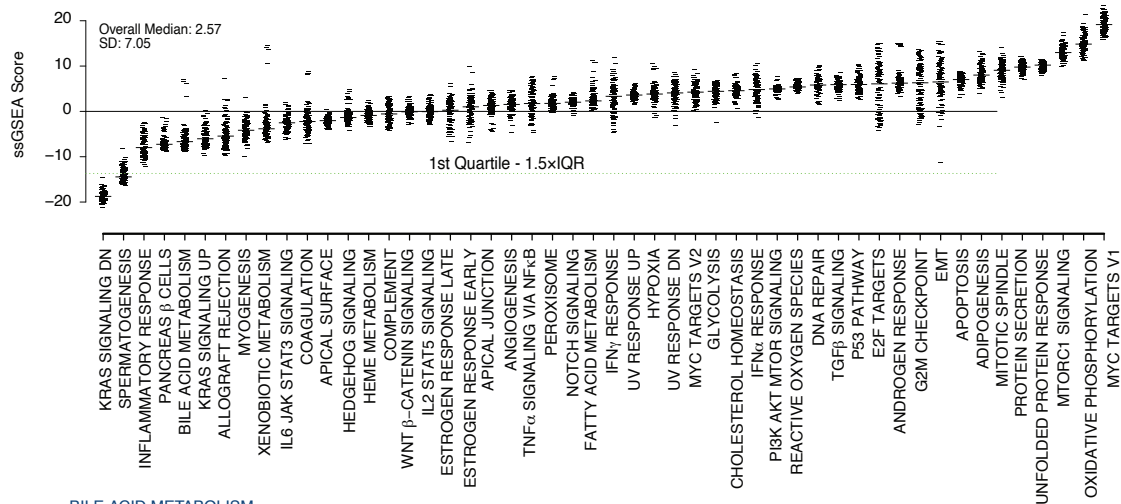

B

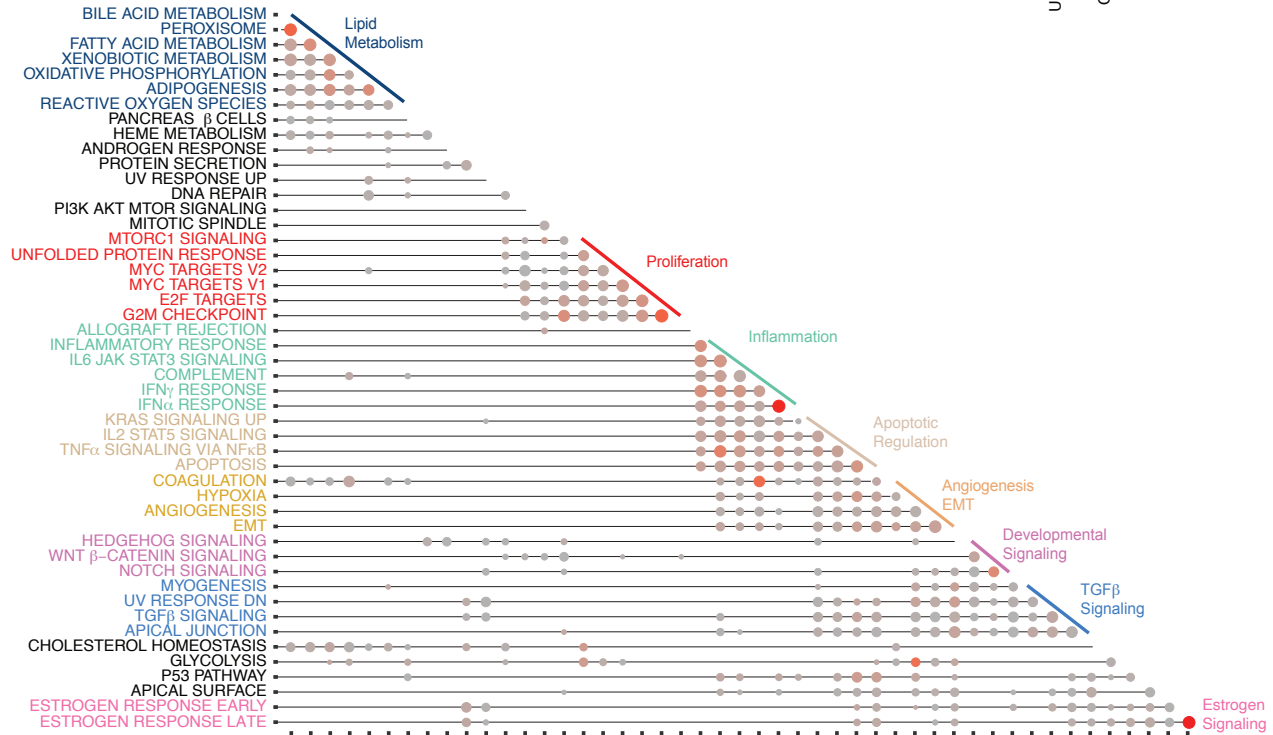

C

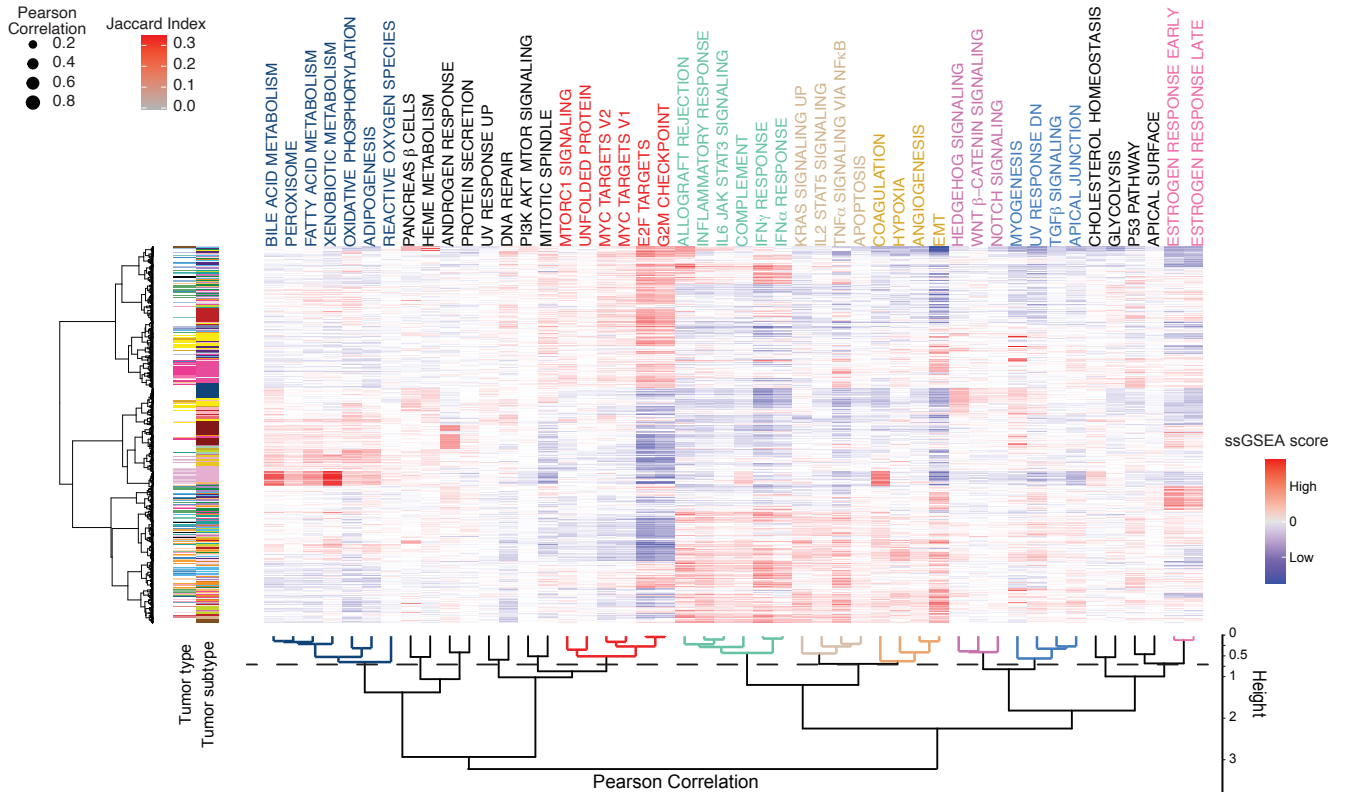

Figure S2

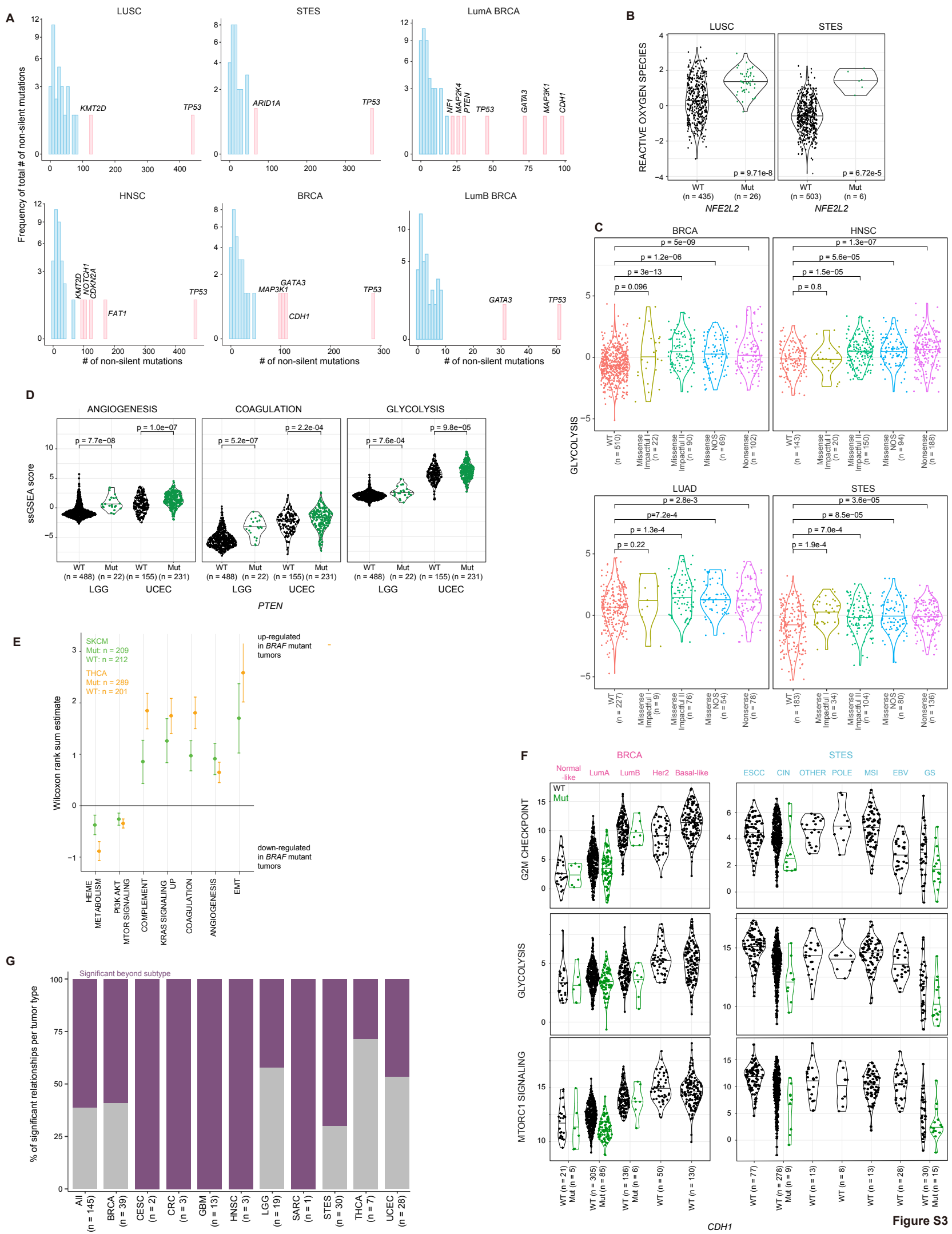

Figure S3

A

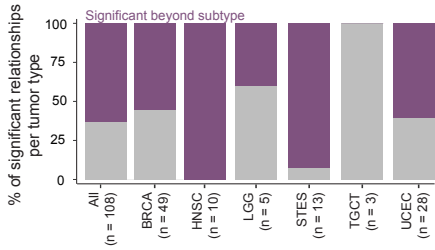

B

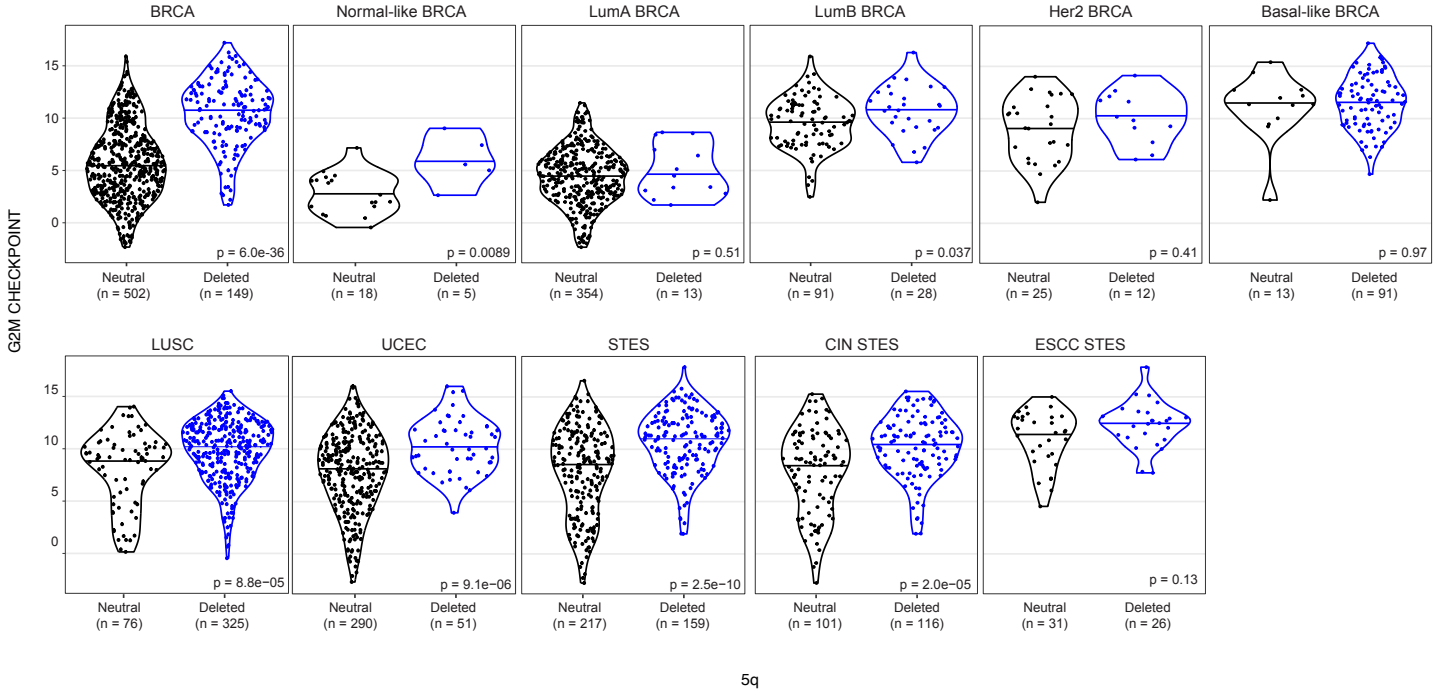

C

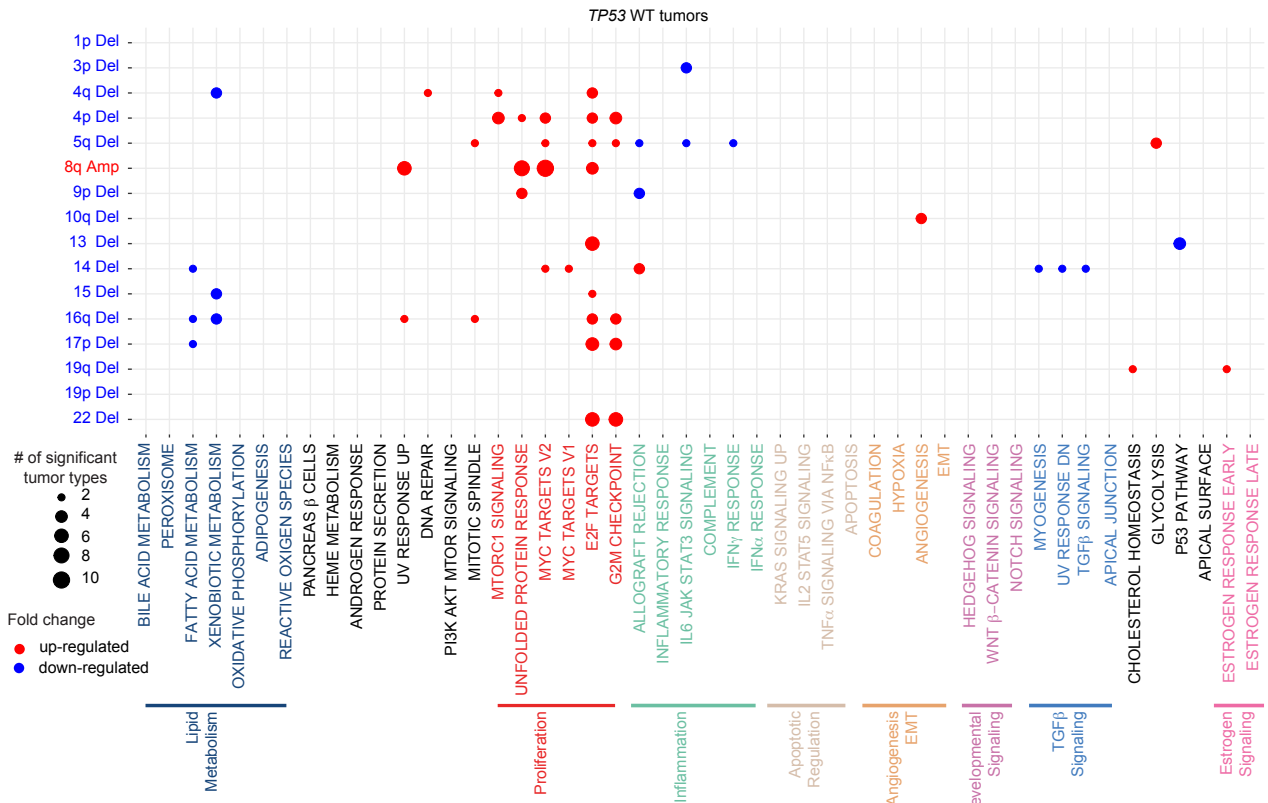

Figure S4

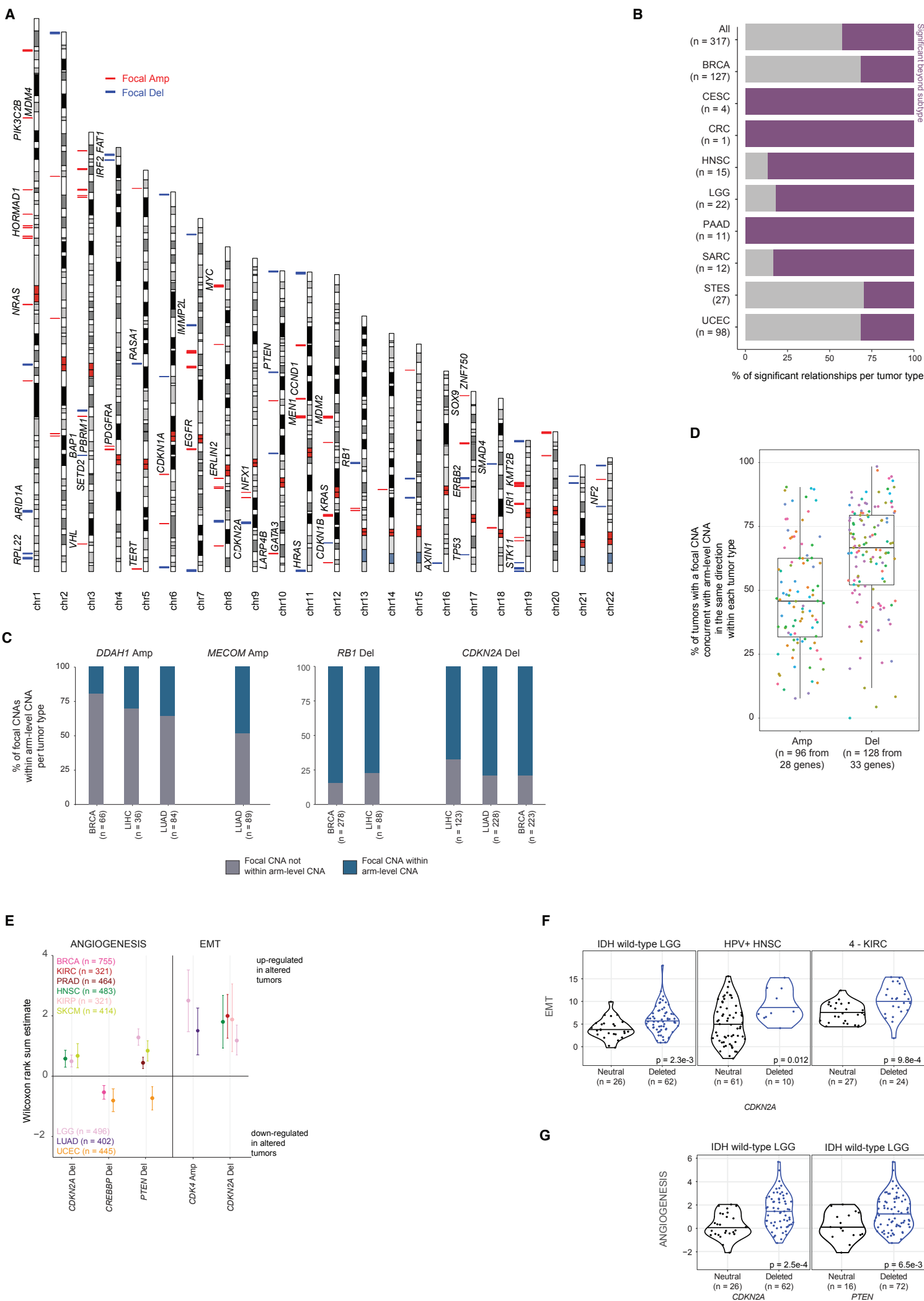

Figure S5

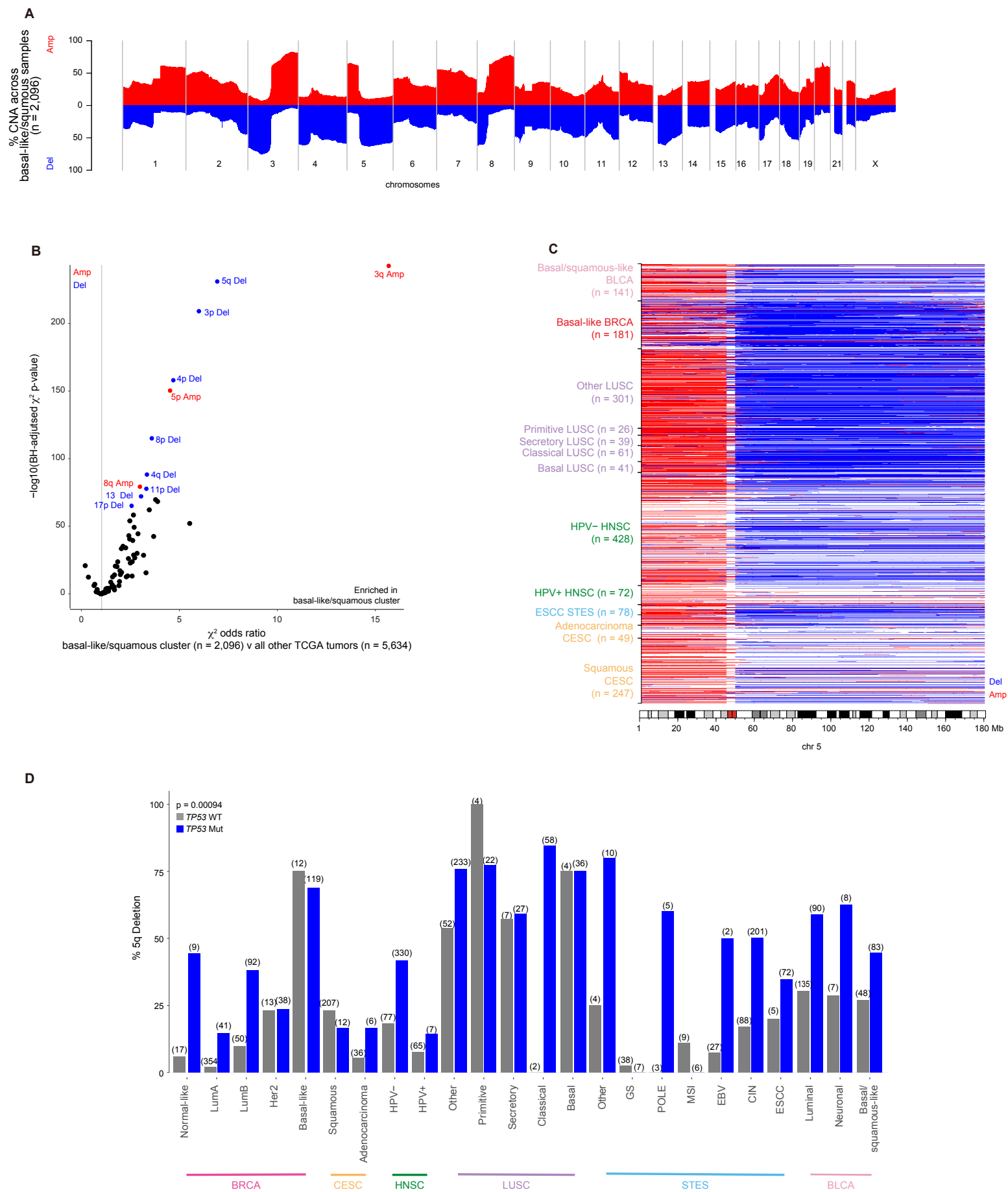

Figure S6

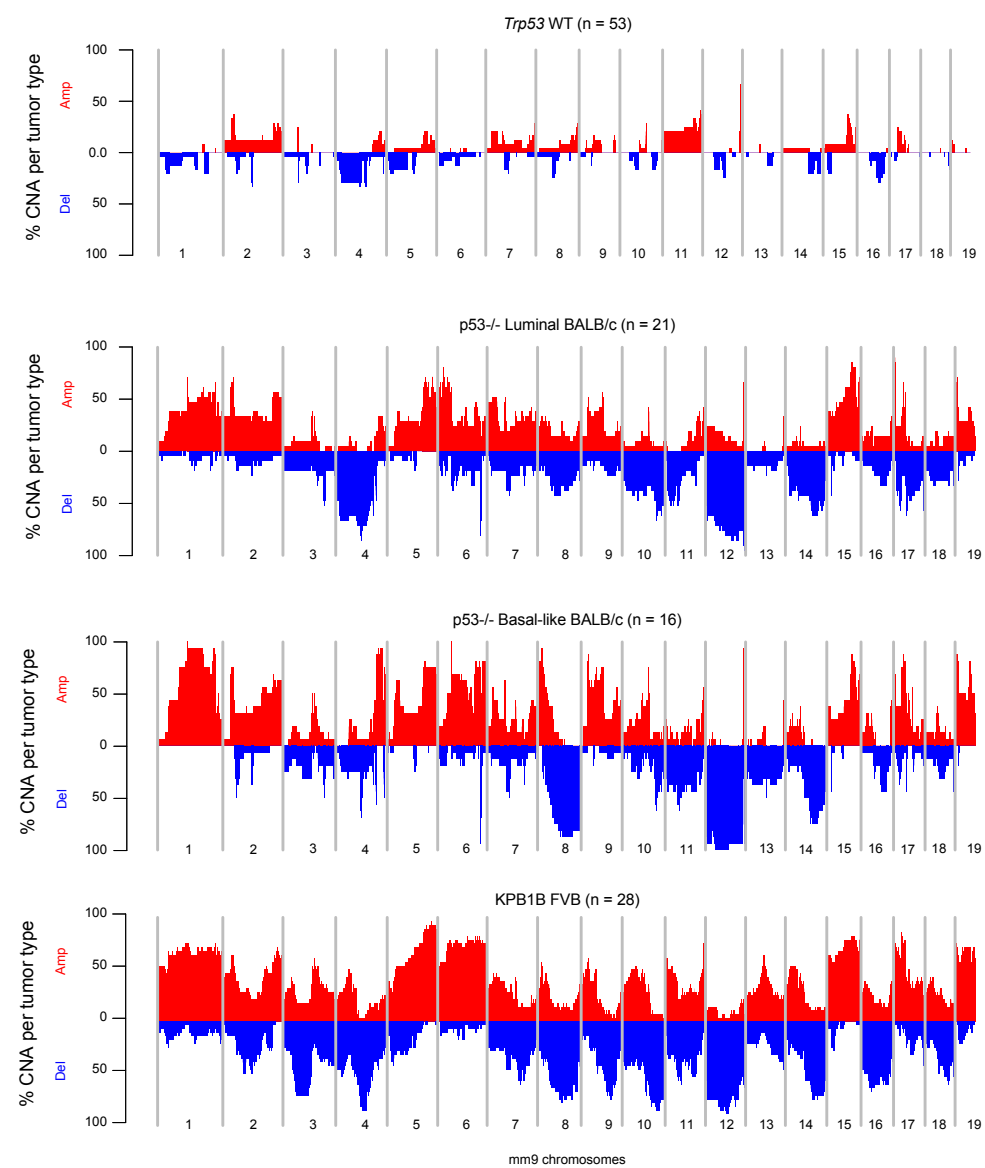

Figure S7

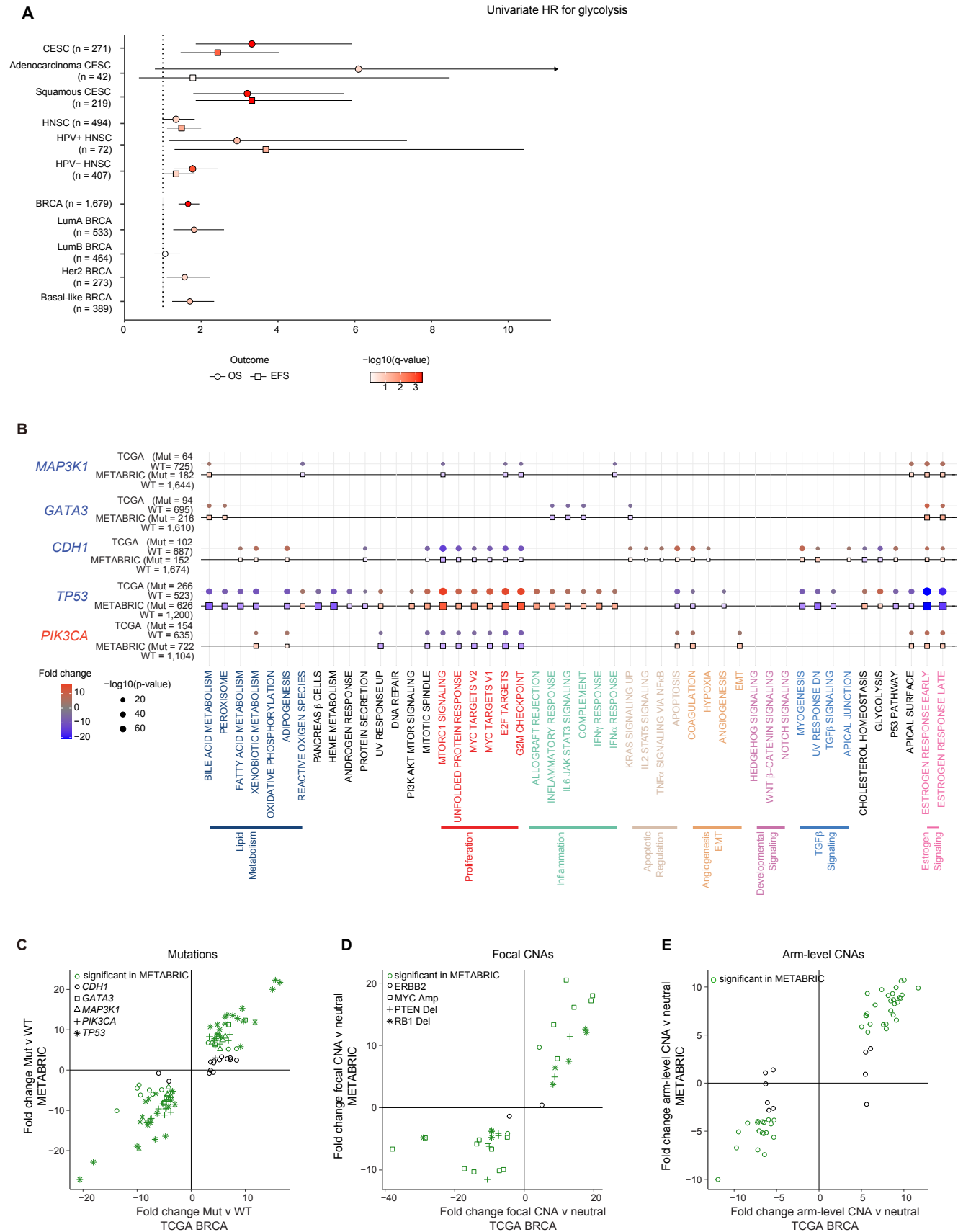

Figure S8

Supplement: Supplementary Figures 1-8 — S1. Methods workflow for pan-cancer analysis of hallmark signatures. S2. Characteristics of hallmark signatures across TCGA and tumor subtypes. S3. Validation of mutation – hallmark signature relationships across TCGA. S4. Validation of arm-level alteration relationship with both hallmark signatures and TP53 mutation. S5. Identification of robust focal alterations and confounding results of mutations and arm-level alterations. S6. Overlap and 5q deletion of basal-like BRCA and squamous tumors compared to HR-driven BRCA. S7. Overview and detailed copy number aberrations of Trp53 wild-type and null genetically engineered mouse models of human breast cancer. S8. Analysis of mutations, hallmark signatures, and multivariate analysis of survival in a large independent primary breast cancer dataset. [file crc-22-0073-s03.pdf]
